# An Implanted Tooth That Can Feel

**DOI:** 10.1002/advs.202520786

**Published:** 2026-03-23

**Authors:** Yaru Cao, Zhenhua Wu, Jiwei Sun, Ke Song, Jiayi Li, Yujin Li, Wenqi Chen, Hanyu Li, Shue Li, Cheng Yang, Silin Liu, Yunyun Han, Bin Su, Yumei Ding

**Affiliations:** ^1^ Department of Neurobiology, School of Basic Medicine, Tongji Medical College Huazhong University of Science and Technology Wuhan P. R. China; ^2^ State Key Laboratory of Materials Processing and Die & Mould Technology, School of Materials Science and Engineering Huazhong University of Science and Technology Wuhan P. R. China; ^3^ Department of Stomatology, Union Hospital, Tongji Medical College Huazhong University of Science and Technology Wuhan P. R. China; ^4^ School of Stomatology, Tongji Medical College Huazhong University of Science and Technology Wuhan P. R. China; ^5^ Hubei Province Key Laboratory of Oral and Maxillofacial Development and Regeneration Wuhan P. R. China; ^6^ Department of Stomatology, Tongji Hospital, Tongji Medical College Huazhong University of Science and Technology Wuhan P. R. China; ^7^ Division of Child Healthcare, Department of Pediatrics, Tongji Hospital Tongji Medical College Huazhong University of Science and Technology Wuhan P. R. China; ^8^ Department of Stomatology Affiliated Hospital of Chifeng University Mongolia P. R. China

**Keywords:** implanted tooth, masticatory perception, piezoelectric conversion

## Abstract

Diverse material species, ranging from gold to ceramic, have been selected to construct dental implants in the past decades. However, existing implants are primarily designed to restore masticatory function yet fail to completely recover the sensory feedback of natural teeth. Deprivation of sensory inputs impairs the perception of food texture and hinders the regulation of chewing force. This defect could result in unnecessary overload, leading to technical complications and biological failures, such as bone loss and temporomandibular joint damage, which remarkably limit their clinical outcomes. To endow the implanted tooth with masticatory perception, herein we demonstrate a 3D‐printed piezoelectric‐core/robust‐sheath implanted tooth can rebuild the sensing feedback, serving as “mechanoreceptors” in converting mechanical chewing force to electrical signals, and up to brain through surrounding alveolar nerves. Working mechanism for the piezoelectric tooth has been revealed by real‐time tracing of neurological activities in the mouse brain in response to simulating occlusal stimulus. Furthermore, more than 90% patients in clinical cases subjectively admitted the rebuilding of their masticatory perception after being implanted the piezoelectric tooth. These findings substantially advance the field of smart implants and herald a promising avenue for medical engineering aimed at enhancing the multi‐functionality of implants in their applications.

## Graphical abstract

To endow the implanted tooth with masticatory perception, herein we demonstrate that a 3D‐printed piezoelectric‐core/robust‐sheath implanted tooth can rebuild the sensing feedback, serving as “mechanoreceptors” in converting the mechanical chewing force to electrical signals, and up to brain through surrounding alveolar nerve system. The working mechanism for the piezoelectric tooth has been revealed by the real‐time tracing of neurological activities in the mouse brain in response to simulating occlusal stimulus. Furthermore, more than 90% patients in clinical cases subjectively admitted the rebuilding of their masticatory perception after being implanted the piezoelectric tooth. These findings substantially advance the field of smart implants and herald a promising avenue for medical engineering aimed at enhancing the multi‐functionality of implants in their applications.

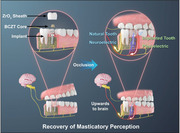

## 1 Introduction

Tooth loss in adults, caused by injury, tooth decay, gum disease as well as aging‐associated physiological degeneration, will bring negative effect on personal appearance and chewing function, thus decreasing the life quality of humans [1, 2]. Dental implants provide a platform to rebuild the masticatory capability of patients, yet fail to completely recover the sensory feedback mechanisms of natural teeth [3, 4]. Such a feedback defect leads to an abnormal sense of taste after implantation, which has been reported in substantial clinical cases [5, 6]. Furthermore, human chewing force is usually overloaded upon non‐sensate dental implants, damaging them as well as surrounding periodontal tissues and bones [7, 8]. Even diverse material species, ranging from gold to ceramic, have been used to construct dental implants [9, 10], their feeling‐free inconvenience has still not been addressed till now.

Masticatory perception of natural teeth originates from the periodontal ligament (PDL) and alveolar bone that enwrap the tooth roots [11, 12]. The PDL contains abundant mechanoreceptors, including Ruffini and Type 3 endings, and periodontal nerves exist within the alveolar bone, capable of generating neuroelectric signals towards the brain in response to humans’ chewing force [13, 14], and these electric signals would contribute to excitation of neural activities in the somatosensory cortex for masticatory perception. Differently, the PDL disappears around the artificial dental implants, indicating the failure of generating neuroelectric signals to the brain. Clinical researchers have devoted extensive efforts to endowing the dental implants with force‐sensitive capability. Nerve regeneration methods based on the bioactive decoration on implanted surfaces [15, 16], as well as artificial PDL by using biomedical engineering, are proposed [17, 18]. However, none of these attempts have effectively rebuilt masticatory perception owing to the limitation of teeth's bio‐complexity.

**FIGURE 1:**
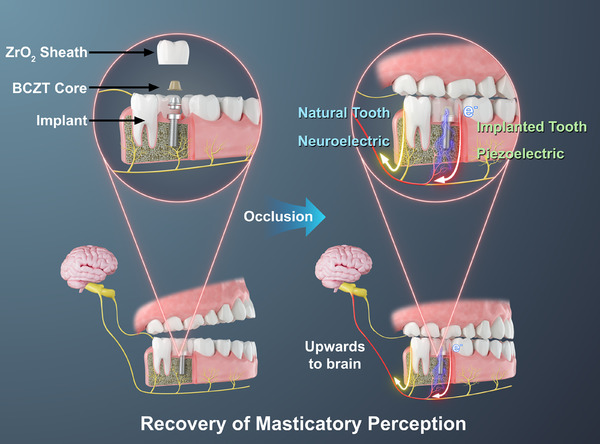
Working mechanisms of a perceivable piezoelectric implanted tooth (PIT). Schematic illustration of exploded view of a PIT during the dental implantation process. The PIT can rebuild the masticatory perception capability for patients, endowing them to feel the food contact in the mouth.

In contrast to the neuroelectric signals generated by mechanoreceptors within the periodontal regions, herein we demonstrate an alternative strategy to restore sensory feedback via a novel dental crown fabricated from piezoelectric materials, termed a piezoelectric implanted tooth (PIT, Figure 1). A piezoelectric‐core/robust‐sheath implanted tooth was 3D‐printed and assembled [19, 20, 21, 22], which can play the “mechanoreceptor” role in converting the mechanical chewing force to electrical signals. The generated signals can be transported through the surrounding nerve system to the brain, rebuilding the sensing feedback loop for masticatory perception. The underlying mechanism for the piezoelectric tooth has been studied by the real‐time tracing of neurological activities in the mouse brain in response to occlusal stimulus, indicating the feasibility of a piezoelectric implanted tooth for masticatory perception. Furthermore, over 90% patients in clinical cases admitted the reconstruction of their masticatory perception after being implanted with the piezoelectric tooth compared with those when equipped with current commercial ceramic dental crowns. This masticatory perception covers a broad range of patients with different clinical backgrounds and presents distinguished sensory sensitivity. Our findings offer valuable insights regarding the implanted tooth that can recover the patients’ masticatory perception, providing opportunities for the fabrication and applications of next‐generation smart dental prosthetics.

## 2 Results and Discussion

### 2.1 Structural Observation of a PIT

**FIGURE 2:**
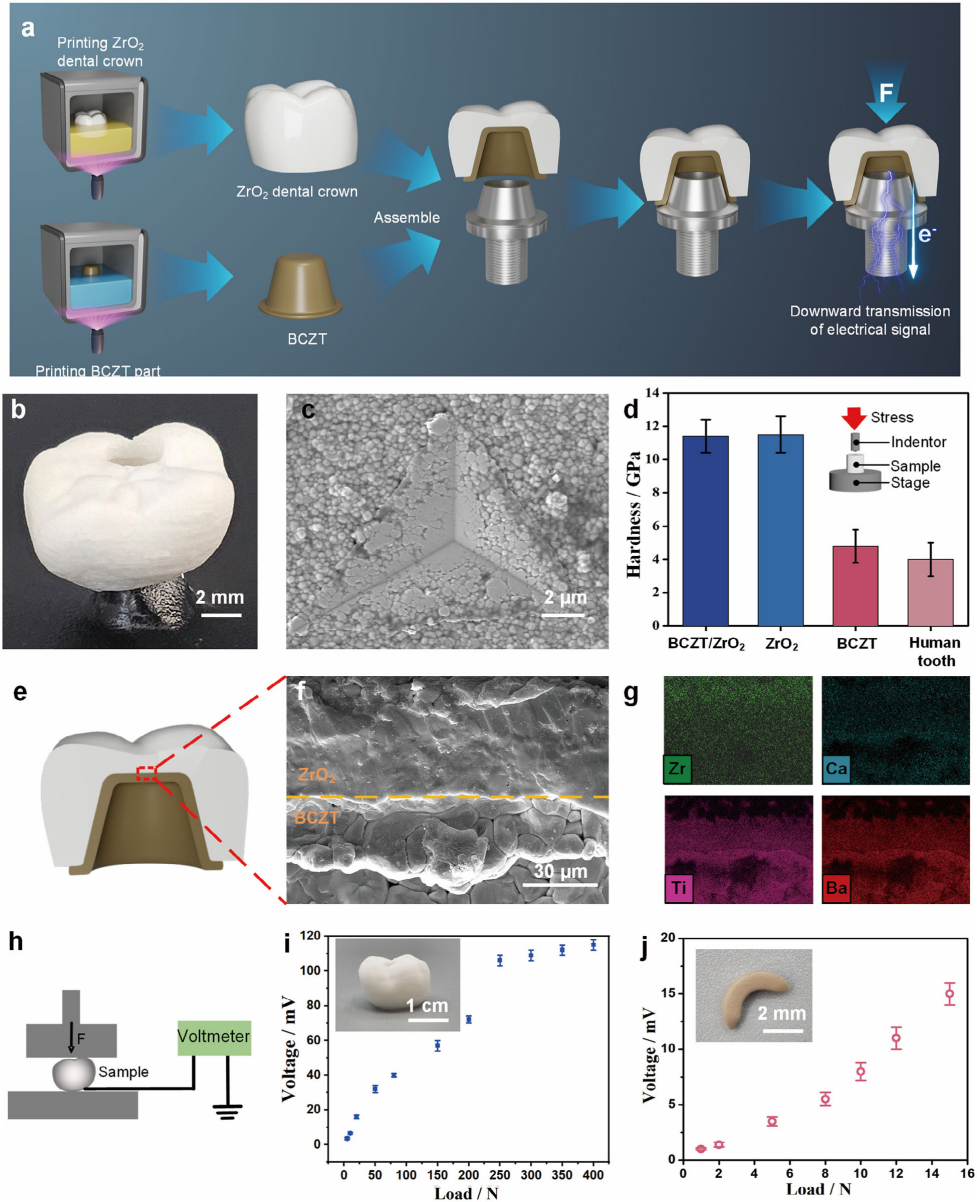
A 3D‐printed PIT that can convert mechanically chewing force to electrical signal. (a) Schematic illustration of fabricating the PIT by two‐step 3D‐printing and then assembly processes. (b) Optical image of a PIT placed on the abutment. (c) Scanning electron microscopy (SEM) image of residual indent of the Berkovich diamond tip on a PIT surface. (d) Hardness comparison of PIT, pure ZrO_2_, pure BCZT and natural human tooth surfaces. (e,f) Cross‐sectional SEM image of the BCZT scaffold. (g) Energy dispersive spectrometer (EDS) mapping images of the ZrO_2_/BCZT interface. (h) Schematic illustration for one‐directional (z) probing of piezoelectric testing. The dependence of output voltage values of a PIT for (i) human and (j) mouse on the applied forces.

The PIT was constructed in two steps through a customized vat photopolymerization 3D printing system [23, 24, 25], which includes the preparation and assembly of a robust dental crown shell and a core with piezoelectric effects (Figure 2a). First, zirconia (ZrO_2_) ceramics with excellent compressive resistance and biocompatibility were selected as the material for personalized customized dental crowns [26, 27, 28]. After light‐curing 3D printing, ZrO_2_ dental crowns with controllable shapes were prepared. For piezoelectric sensing core materials, due to the requirements for mechanical properties, biocompatibility, and piezoelectric conversion performance, a lead‐free and biosafe barium calcium zirconate titanate piezoelectric type (BCZT) material was selected as the core of piezoelectric dental implants (PIT) [29, 30, 31]. Notably, through classical in vitro experiments (MTT assay and Live/Dead staining), the BCZT was highly biocompatible and biosafe for potentially clinical applications (Figure S1). Then, the structure customization of BCZT cores can be achieved through UV‐curable printing and post‐processing. Considering the mechanical robustness of assembled PIT, the ZrO_2_ dental crown and BCZT were further solidified by a BCZT composite paste. The preparation details can be found in Experimental Section and Figures S2 and S3.

Figure 2b shows the optical image of a PIT (8 mm in diameter and 7 mm in height) placed on the abutment. It resembles a patient volunteer's tooth structure in terms of white appearance and surface roughness. Top‐view scanning electron microscope (SEM) observation of a PIT shows that the ZrO_2_ grains coalesce well after the 3D printing and then sintering process (Figure 2c), leading to a mechanically robust top. Figure S4 shows SEM images of the printed BCZT part. The BCZT particles on the surface and in the cross‐section of the printed part are closely combined, enabling them to maintain mechanical stability during the stress‐polarization transition process [32, 33, 34]. The microscopic mechanical properties of the PIT surface have been comparatively studied by nanoindentation (NI, Figure 2c). No obvious material accumulation or depression was observed at the edge of the indentation, indicating that ZrO_2_ ceramics mainly undergo elastic deformation under this load, accompanied only by a small amount of plastic flow, demonstrating the excellent pressure‐resistance of the ZrO_2_ sheath. The hardness of PIT, pure ZrO_2_, pure BCZT, and natural human tooth has been compared (Figure 2d). The hardness of PIT is 11.8 ± 0.7 GPa, which is higher than that of human tooth (3.8 ± 0.6 GPa) [35, 36]. This value can guarantee the PIT to bear the chewing force of humans in daily life. The cross‐sectional morphology of ZrO_2_/BCZT interface was characterized by SEM, with results presented in Figure 2e,f. It is clearly found that ZrO_2_ and BCZT layers closely contact with each other without obvious cracks. The multi‐elements EDS mapping images of ZrO_2_/BCZT interface further confirmed the existence of Zr, Ca, Ba, and Ti, indicating well coalesce of two kinds of ceramics (Figure 2g).

### 2.2 Piezoelectric Capability of a PIT

Furthermore, the piezoelectric properties of different PITs (including samples for humans and mice) were investigated after electrical polarization (Figure 2h–j). Under the induction of different applied pressures, both PITs show trends of increasing electrical output with the increase of pressures (Figure 2i,j). Generally, the occlusal force of a human tooth can reach ∼250 N [37, 38, 39]. Under this pressure, the output voltage of the PIT is 108 ± 5 mV. In addition, the long working life and reliability of the PIT were tested (Figure S5). The results show that its voltage output performance remains stable even after 10 000 periodic compression and release cycles. Besides the PIT for human, we also prepared a 3D‐printed PIT for experimental mice (Figure 2j), which will be then studied in the real‐time tracing of neurological activities in their brains. The smaller PIT sample showed a similar piezoelectric capability, with 15 ± 2 mV value at the applied force of 15 N (similar to the value of the mouse tooth's chewing force) [40, 41].

### 2.3 Establishment of the Model for Neuroimaging of Mice Receiving Masticatory Information Through PIT

**FIGURE 3:**
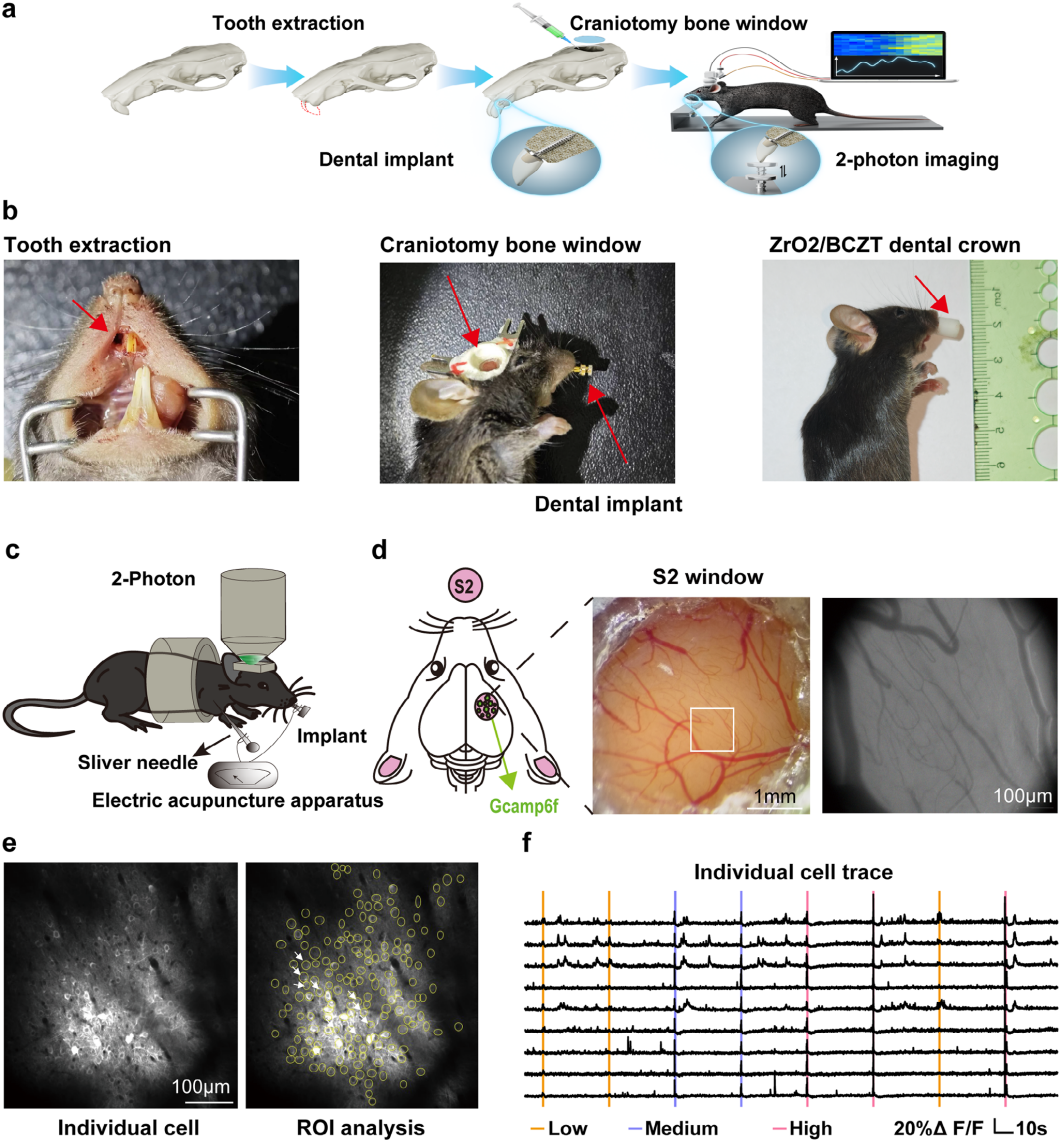
Real‐time tracing of neutral activities in response to occlusal stimulus using two‐photon Ca^2+^ imaging of S2 in head‐fixed mice. (a) Schematic illustration of procedures of dental implantation and image capturing. Optical photographs of (b) experimental mice after extraction of incisors, experimental mice after dental implantation and cranial window operation, and dental implants equipped with perceptive tooth crown. (c) Scheme of image capturing on mice using two‐photon microscopy. (d) The figure presents an illustration and a photograph of the cranial window engineered for imaging the secondary sensory cortex (S2). (e) The panels in left shows in vivo two‐photon imaging of GCaMP6f fluorescence recorded from S2 neurons in a mouse in the occlusal stimulus, the panels in right show the regions of interest (ROI). (f) Representative neural response in S2 regions during loading of occlusal stimulus at different levels, the black traces illustrate the GCaMP6f fluorescence transients (ΔF/F, ∆F/F=F_t_‐F_0_/F_0_, with the baseline established as the 10 s before the first stimulation. Where F_0_ is the average between the 25th and 80th percentiles of the fluorescence signal at the baseline 10 s before the start of the first stimulus, and Ft is the fluorescence signal recorded in real time), capturing the activity profiles of a representative sample of ROIs previously identified in this field of view (FOV) depicted in (e).

To observe the natural feedback of occlusal perception by using the PIT, we constructed a novel neuroimaging experimental system in mice (Figure 3a). A total of 8 mice were included for further operations. Briefly, the right incisor of each mouse was extracted after general anesthesia (Figure S6), followed by the placement of a customized titanium implant into the extracted site (Figure 3b). A PIT dental crown (3 mm in diameter and 5 mm in length) was then installed onto the implant to substitute for the lost incisor. Experimental mice could continue living, as HE sections of several internal organs indicated that the series of surgical operations was biosafe (Figure S7). Upon biomimetic mechanical loading on PIT, in vivo two‐photon microscopy was employed to record real‐time neural activities in the somatosensory cortex (S2) evidenced by changes in fluorescent signals emitted by Ca^2+^ massagers GCaMP6 (Figure 3c, Figure S8) [42, 43, 44].

Following the above scheme, incisors of mice were successfully replaced by PITs to simulate clinic situations, along with operations on brain regions for further head fixation and signal capture. PIT was expected to generate and deliver electric signals to tightly‐contacted dental implants when confronted with external mechanical loading (Figure 3a). S2 was imaged in head‐fixed mice through a glass window above the brain cortex (Figure 3d). Regions of interest (ROIs) were extracted for the calculation of normalized changes in fluorescence (ΔF/F) (Figure 3e). When mice were trained to proficiency, we observed significant increases in ΔF/F values once mechanical loadings at different levels were applied, which could be stably repeated (Figure 3f). Thus, we successfully placed PIT on natural tooth‐extracted sites to simulate clinic scheme and captured obvious neural activities in response to mechanical cues, proving that PIT was capable of transforming occlusal loadings into neural perception.

### 2.4 PIT Rebuilds Neural Activities in Somatosensory Cortex in Response to Masticatory Loading

As natural tooth has been reported to distinguish variant degrees of occlusal loadings (High‐level: ∼10N, Medium‐level: ∼6N, Low‐level: ∼3N) for feedback regulation [45, 46, 47], we further evaluated whether PIT could achieve this exquisite function. Mechanical loading‐induced neural activities from a natural tooth were acquired and set as a positive control to analyze whether PIT and ZrO_2_ crown could reproduce the mechanical perception potential of the natural tooth. Dynamic Ca^2+^ signals from S2 neurons in ZrO_2_, natural tooth, and PIT group were recorded upon different mechanical loadings. We mainly focused on traces of S2 neural activities and estimation of whether differential neural responses were present associated with low, medium, and high mechanical loadings. Finally, mechanical loading‐induced neural activities in ROIs from a total of 1250, 1402, and 1195 neurons were collected in ZrO_2_, natural tooth and PIT group, presenting a concordant response pattern (Figure S9).

**FIGURE 4:**
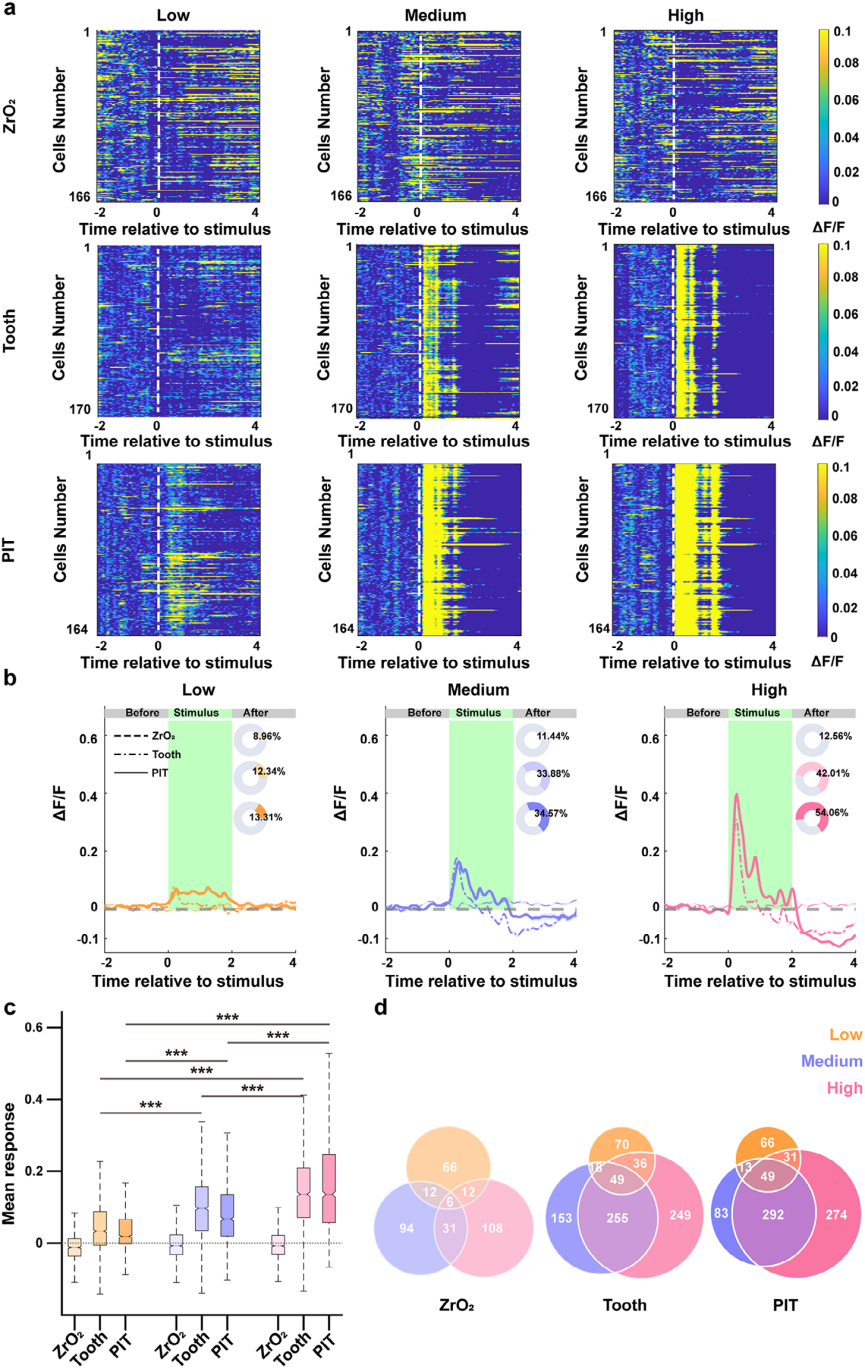
Differential neural activities in response to different levels of occlusal stimulus. (a) The top row of panel A represents the heatmap of neural activity of each S2 neuron in response to different occlusal loads in a representative mouse from the ZrO_2_ group. The middle row of panel A represents the heatmap of neural activity of each S2 neuron in response to different occlusal loads in a representative mouse from the natural tooth group. The bottom row of panel A represents the heatmap of neural activity of each S2 neuron in response to different occlusal loads in a representative mouse from the PIT group. (b) Trail‐averaged neural response amplitude of recorded all S2 neurons during occlusal stimulation at different levels and neural response cell fraction from the ZrO_2_, natural tooth, and PIT groups. (c) Population‐averaged Ca2+ response in (a) from the ZrO_2_, natural tooth, and PIT groups. Statistical analysis of (d) neural response number in trials with different levels of occlusal stimulus from the ZrO_2_, natural tooth, and PIT groups. Data analyzed by (c, (ZrO_2_ (*n* = 7 mice), tooth (*n* = 7 mice), PIT (*n* = 8 mice)) two‐sided two‐way repeated measures ANOVA for analysis with post‐hoc Bonferroni comparisons. Data analyzed by (d, (ZrO_2_ (*n* = 7 mice), tooth (n = 7 mice), PIT (*n* = 8 mice)) Wilcoxon signed‐rank test to define responsive cell population. Data are presented as (b, mean ± s.e.m. or (c, box plots (center line, median; box limits, upper and lower quartiles; whiskers, 1.5 × interquartile range).

As expected, all levels of mechanical loadings could only evoke weak and random neural activities in ZrO_2_ group. In comparison, multiple mechanical loadings could evoke rapid S2 neural responses in natural tooth and PIT group evidenced by significant increasing tendency of neural activities (Figure 4a, Figures S10–S12). High‐level stimulus produced the highest peak level of ΔF/F, followed by medium and low level stimulus sequentially in these two groups. Interestingly, neural response induced by low‐ and medium‐level stimuli returned to basal levels as soon as the stimulus ended, whereas the neural response to high‐level stimulus was delayed and returned back to basal level after the stimulus ceased (Figure 4b). Variant levels of mechanical loading elicited different peak levels as well as duration times of S2 neural activities when using natural tooth and PIT, suggesting that PIT could recover the masticatory perception with a similar pattern to that of natural tooth. Neural responses from different levels of stimulus could be repeated among trials, and S2 neurons represented remarkably enhanced mean response intensity during high‐level stimulus compared with other levels of occlusal stimulus in the natural tooth and PIT group. On the contrary, mice with ZrO_2_ crown failed to distinguish different levels of occlusal forces, evidenced by unchanged and weak neural signals (Figure S13a, Figure 4c). When receiving repeated randomly‐distributed occlusal trials, the response cell fraction exhibited as 13.31%, 34.57%, and 54.06% for low, medium, and high levels of occlusal stimulus in PIT group, similar to that in the natural tooth group (12.34%, 33.88%, and 42.01% respectively) (Figure 4b). Furthermore, a substantial overlap was observed among these responsive neurons in PIT and natural tooth groups (292 and 255 neurons, respectively) (Figure 4d). This suggested that increase of occlusal stimulus could recruit more neurons into S2, activate them for transmitting and processing sensational information, which reproduced neural regulatory pattern associated with natural teeth [48, 49].

Additionally, correlation analysis of cell and trial showed that stronger mechanical loading led to higher mutual communication and synchrony among neurons, as evidenced by the highest correlation coefficients in the high‐stimulus group (Figure S13b,c), suggesting that with an increase of occlusal loadings, neurons in S2 exhibited a tighter functional interaction for improved perception and information processing. In summary, differential changes in total response intensity, response ratio as well as functional connection of S2 neurons associated with multiple occlusal loadings proved that PIT was capable of rapid perception and precise discrimination of masticatory information, recovering the perception capability and discrimination pattern of natural tooth to a great extent.

### 2.5 Potential Clinic Application of PIT

**FIGURE 5:**
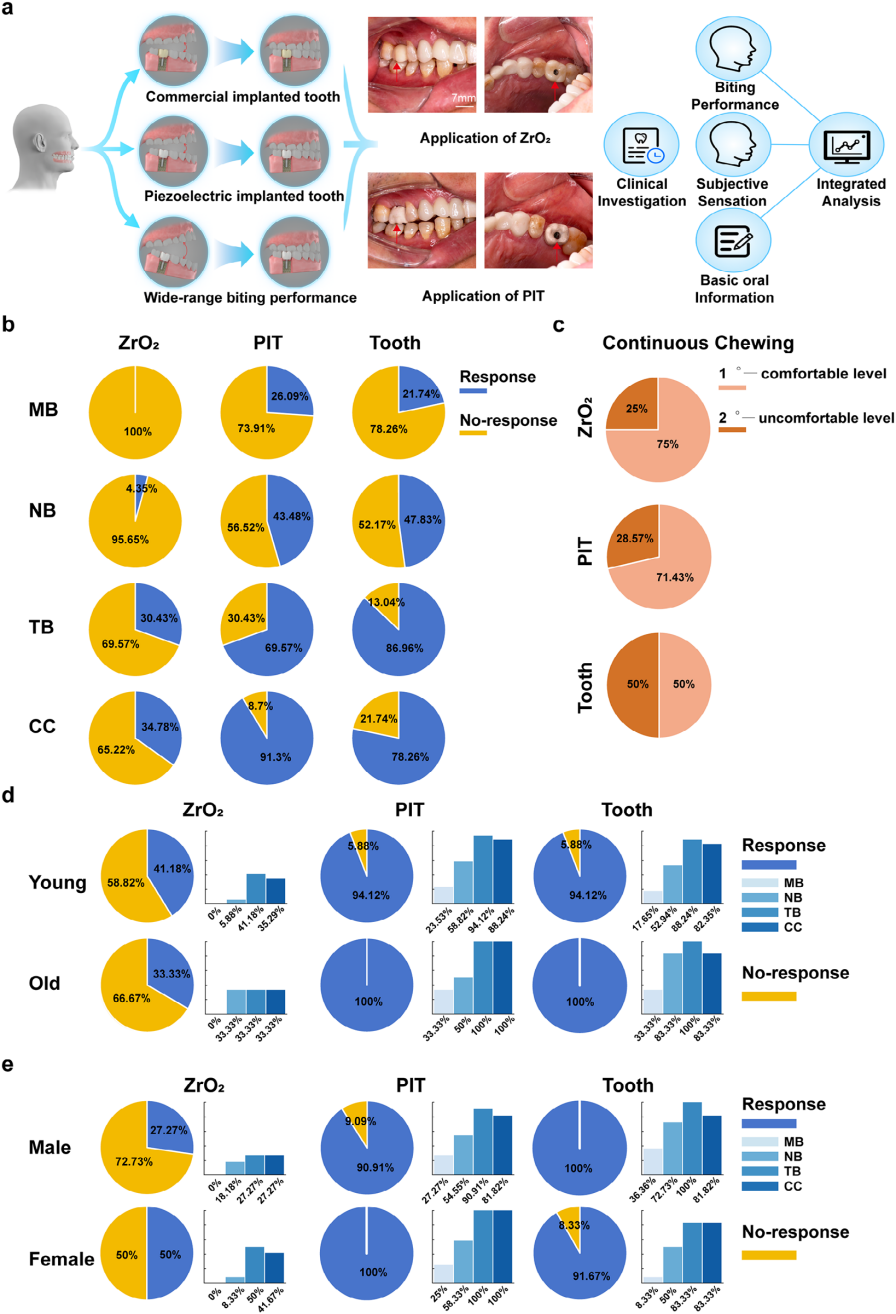
Clinical test of application of perceptive tooth for occlusal sensation recovery. (a) Schematic diagram of clinical test for masticatory sensation using ZrO_2_ dental crown, PIT dental crown, and natural tooth. Red arrows indicate test of PIT). (b) Subjective response status of patients under different levels of occlusal loadings when using ZrO_2_ dental crown, PIT dental crown, and natural tooth, respectively. Biting performances are divided into 4 types: mild biting (MB), normal biting (NB), tight biting (TB) as well as continuous chewing (CC) to distinguish occlusal force and pattern. (c) Response intensity of patients using ZrO_2_ dental crown, PIT dental crown and natural tooth when carrying out continuous chewing performance. (d) and (e) Integrated analysis of the relationship between age (d), gender (e) and response rate to different levels of occlusal loadings.

Finally, we made efforts to confirm the clinical potential of PIT with the help of volunteers in need of dental implantation, who were gathered in the Department of Stomatology, Union Hospital, Tongji Medical College, Huazhong University of Science and Technology. A total of 23 volunteers meeting key inclusion criteria were recruited for information collection and clinical examination with their personal consents (Table S1). In detail, volunteers having received stable dental implantation and ready for phase 2 prosthetic operation were first consulted for the acquisition of their baseline demographics and disease characteristics (Figure 5a, Figure S14). The median age of enrolled volunteers was 44, in which there were 12 males and 11 females included. The experiment was divided into 3 groups: installation of commercial implanted tooth, installation of piezoelectric tooth and a natural tooth on the opposite side of the implanted tooth for biting and chewing test.

As expected, the control normal tooth group exhibited a high‐level response rate during all four types of biting experiments: tight biting (TB, ∼200 N), normal biting (NB, ∼100 N), mild biting (MB, ∼50 N), and continuous chewing at ∼100 N (CC) [50]. However, only a small portion of patients responded to biting experiments (3 in normal biting and 9 in tight biting) when using ZrO_2_ crown, which could be explained by compensatory sensation through neighboring normal teeth and alveolar bone [51, 52, 53]. In comparison, application of PIT resulted in a significant improvement in subjective masticatory sensation upon occlusal loadings, with the response rate amounting up to 25.93%, 40.74%, and 66.67% in mild, normal, and tight biting, respectively (Figure 5b). Along with the strengthening of biting force, the response rate of PIT exhibited an increasing tendency (from about 25% to 67%), simulating the sensory pattern of a normal tooth [54, 55], which indicates that application of PIT can recover natural masticatory discriminating capability to some extent. Specifically, the response ratio of PIT during continuous chewing was above 80%, even leading up to that of control normal teeth, indicating that PIT might generate superior and stable perception during the real food uptake process, which requires continuous chewing [56, 57].

In addition, PIT showed above 70% comfortable‐level intensity (sensation without stimulus or pain) in all four biting experiments, suggesting that its piezoelectric conversion potential is adequate for occlusal perception while maintaining biocompatibility and bio‐comfortability (Figure 5c). In perspective of clinical information, we first divided volunteers into young (30–50) and old (>50) groups, and observed that while a small part of volunteers reported occlusal perception of ZrO_2_ crown (42% and 25%), more than 70% volunteers reported PIT responses when biting, similar to that of normal tooth, confirming the feasibility of PIT in all age groups (Figure 5d). Analogously, male and female volunteers also reported similar PIT response rate, supporting its prevalence in all gender groups (Figure 5e). However, limited by the feasibility of the current clinical operation, only subjective feedback from volunteers was collected to support our concept. Objective measurements, brain CT scanning, for example, were required for further exploration. Taken together, the above clinical exploration proved that PIT possessed excellent potential in clinical application for reconstruction of masticatory sensation as well as discrimination.

## 3 Conclusion

In summary, we have successfully engineered a manufacturing and assembly pathway to fabricate piezoelectric dental implants. The designed artificial tooth achieves conversion of occlusal force into electrical signals up to the sensory cortex, shedding light on a solution to the critical clinical dilemma of masticatory perception post tooth implantation.

## 4 Experimental Sections

### 4.1 Preparation of BCZT Powders

According to the element ratio of BCZT (0.5(Ba_0.7_Ca_0.3_) TiO_3_‐0.5Ba (Zr_0.2_Ti_0.8_) O_3_), weigh the high‐purity raw materials of barium carbonate (99.5%, China National Pharmaceutical Group Corporation, Shanghai), calcium carbonate (CaCO_3_; 99.5%, China National Pharmaceutical Group Corporation), zirconium dioxide (ZrO_2_; 99.5%, China National Pharmaceutical Group Corporation), and titanium dioxide (TiO_2_) respectively. Mix the powders and calcine them at 1250°C for 4 h. Then grind them in anhydrous ethanol (CH_3_CH_2_OH >99.5%, Sinopharm) and finally sieve to obtain high‐purity BCZT powder with an average particle size of 1.2 µm.

### 4.2 Fabrication of BCZT Blocks

UV printing resin containing 40 vol% BCZT powder was prepared. The green body was printed by exposing the resin to ultraviolet light (λ = 405 nm) using a commercial digital light printer (AutoCera, Beijing 10dim Tech. Co., Ltd., China). Printing parameters: Single‐layer curing thickness 25 µm, exposure time 8 s. The printed green bodies were ultrasonically rinsed with acetone and ethanol, respectively to remove the uncured resin. Then, the printed green bodies were heated in argon gas at a heating rate of 0.2°C/min at 600°C for 4 h to remove organic matters. The final BCZT blocks were obtained by sintering the degreased green bodies in air at 1500°C for 3 h, at a heating rate of 3°C/min.

### 4.3 Fabrication of ZrO_2_ Dental Crown

UV printing resin containing 40 vol% 3Y‐ZrO_2_ powder was prepared. The green body of ZrO_2_ dental crown was printed by exposing the resin to ultraviolet light (λ = 405 nm) using a commercial digital light printer (AutoCera, Beijing 10dim Tech. Co., Ltd., China). Printing parameters: Single‐layer curing thickness 25 µm, exposure time 8 s. The printed green body was ultrasonically rinsed with acetone and ethanol, respectively to remove the uncured resin. The green body was heated in argon gas at a heating rate of 0.2°C/min at 650°C for 5 h to remove organic matters. Finally, the ZrO_2_ dental crown was obtained by sintering the degreased green body in the air at 1500°C for 3 h.

### 4.4 Fabrication of PITs

The human PIT was fabricated by assembling the ZrO_2_ dental crown and the BCZT block. Specifically, the ZrO_2_ dental crown and the BCZT block were assembled by pasting a thin layer of UV printable resins containing 40 vol% BCZT powders. Then, the ZrO_2_/BCZT composite PIT was sintered in air at 1500°C for 3 h, at a heating rate of 3°C/min, to obtain the final human PIT. The mouse PIT was fabricated via a similar procedure yet in a smaller size.

### 4.5 Polarization of PITs

The polarization of the human PIT was carried out by poling for 10 s in 25°C silicon oil under a DC electric field of 3 kV/mm. Before polarization, the upper and lower ends of the BCZT part of the human PIT were coated with conductive silver paste and sintered at 500°C for 1 h. The polarization of the mouse PIT was as the same of the polarization process of the human PIT. The room‐temperature piezoelectric coefficient *d*_33_ of the obtained samples was measured by using the quasi‐static piezoelectric constant tester (ZJ‐3AN, Institute of Acoustics, Beijing, China) after aging for 24 h. Before these PITs were clinically applied, the conductive silver paste was repeatedly ultrasonically washed with ethanol to ensure safety.

### 4.6 Artificial Saliva Preparation Method

1.286 g of NaCl, 1.864 g of KCl, 0.221 g of CaCl_2_·2H_2_O, 0.140 g of NaH_2_PO_4_·2H_2_O, and 1.680 g of NaHCO_3_ have been added in 800 mL of deionized water under stirring at 300 rpm. Ensuring complete dissolution of each component before adding the next one. Heating to 37°C and stirring at this constant temperature for 30 min. Adjusting the pH to 7.40 ± 0.05 using 0.1 mol/L NaOH or HCl, then cooling to 25°C and adding deionized water to 1 L.

### 4.7 Piezoelectric Characterization

Responsive electrical signals of the poled PITs were tested under continuous compression/recovery and applied pressure stimulation. Continuous cyclic loads were applied to the PITs using an electronic dynamic static‐fatigue testing machine (E1000, Instron‐Division of ITW Ltd., MA, USA). The voltage output signals during cyclic compression/pressure process were recorded using a data acquisition and digit multimeter system (DMM 7510, Tektronix, Beaverton, OR, USA).

### 4.8 Characterization

The microstructure of the ZrO_2_, BCZT powders, and the printed PITs were observed via scanning electron microscope (SEM, JSM‐7600F, JEOL, Japan). Nanoindentation (NI) tests were carried out by an Agilent G200 nano indenter equipped with a continuous stiffness measurement (42) (CSM) module. The samples were stored and the data were obtained at a relative humidity (RH) of 35%, and the temperature was 25°C. A PIT sample were indented on the top. The indented depth for all samples was 2000 nm. The final elastic modulus and hardness data were obtained by calculating the mean value between 200–400 nm. 10 points were indented for each specimen and 3 different specimens were tested for each material.

### 4.9 Animals

The study involved the execution of all experimental procedures as per the protocols endorsed by the Institutional Animal Care and Use Committee at Huazhong University of Science and Technology (IACUC Number:4462). The study employed male C57BL/6 wild‐type mice aged between 2 and 4 months. Post‐surgical recovery involved single‐cage rearing. Environmental conditions were standardized to include a 12‐hour light/dark cycle (8:00 am–8:00 pm light, 8:00 pm–8:00 am dark), a temperature of 23 ± 2°C, and relative humidity maintained within the 45%–75% range. Experiments were conducted only while the light cycle was active.

### 4.10 Surgery for Craniotomy

To image calcium in mouse S2 neurons in vivo, we first stereotaxically located the S2 region (AP −0.82 mm, ML −4.2 mm, DV −0.3 mm, per the Allen Mouse Brain Atlas, DV was localized using the dura mater as the zero point). A craniotomy was then performed, creating a circular window to expose the S2 area. We injected the virus (rAAV2/9‐CamKII‐GCaMP6f, 50 nL, 10^10^ VP/mL) using a glass micropipette driven by a pneumatic pump. Following this, a 4 mm diameter glass coverslip was implanted, and the window was sealed with Pattex cyanoacrylate. Finally, a custom titanium head mount was secured to the skull with dental cement to enable stable head fixation (C&B Super‐Bond, BearDayton).

To ensure the successful completion of cranial window surgery. First, dexamethasone (5 mg/kg) was administered subcutaneously to all mice 24 h prior to the operation to mitigate the risk of brain swelling. Then, anesthesia was induced via an intraperitoneal injection of 1.25% tribromoethanol, dosed at 0.2 mL per 10 grams of body weight. Next, a local anesthetic, 1% lidocaine (0.02 mL per subject), was given subcutaneously just before the skin incision was made. Once the mice reached a profound state of anesthesia, they were secured in a stereotaxic frame (from RWD Life Science) to commence the surgical intervention. Throughout the duration of the surgery, body temperature was kept constant at 37 degrees Celsius using an electric heating pad with temperature feedback control.

### 4.11 Surgery for Dental Implantation

To place dental implants into the alveolar bones of mice, 8‐week‐old mice were anesthetized, extracted the right maxillary incisors, immediately implanted the customized mental implant screws into their alveolar fossa, and then screwed in the matching dental crowns respectively.

After surgery, mice recovered fully on a heating pad before returning to the animal room. For post‐operative pain and inflammation management, they received subcutaneous injections of tolfenamic acid (0.5 mg/kg, Tolfedine, Vetoquinol) for three days and enrofloxacin (0.5 mg/kg, Baytril, Bayer) for seven days.

### 4.12 Installation of Dental Crowns

When dental implants were successfully placed and integrated into the alveolar bone, different 3D‐printed dental crowns (pure ZrO_2_ crown and double‐layered ZrO_2_/BCZT crown, named PIT) were installed and fixed onto implants to simulate clinical prosthetic operation for further experiments.

### 4.13 Simulation of Occlusal Behaviors

To study the role of different dental crowns in the sensation of occlusal information, a custom‐made spring‐piston type device was fabricated to simulate occlusal behavior by controllable contact with implanted crowns. To mimic different types of occlusal forces, compression of springs was set at variant levels to generate forces of low, medium and high intensities, which were labelled as 1, 2, and 3, respectively. During the whole test, mice were treated in a pseudo‐random manner, with the sequence being (1, 1, 2, 2, 3, 3, 1, 3, 1, 2, 2, 3, 1, 2, 3).

### 4.14 Histological Staining

For histological staining of brain samples, mouse brains were fixed overnight in 4% PFA, then dehydrated in 30% sucrose for 48 h. The sucrose concentration was reduced to 20% for another 48 h. Heads were cryosectioned at 50 µm coronal thickness (Leica CM1950), and brain slices were collected. Finally, slices were imaged at 10× (NA 0.4) using an Olympus VS120 slide scanner.

For hematoxylin and eosin (H&E) staining of the heart, liver, spleen, lung, and kidney. Tissue samples were dehydrated, embedded in paraffin, and sectioned into 4 µm thick slices. H&E staining was performed following routine procedures. Briefly, tissue sections were deparaffinized and rehydrated with xylene, 100% ethanol, 95% ethanol, 80% ethanol, and deionized H_2_O in sequence. Subsequently, the sections were stained with hematoxylin and destained with acid ethanol, followed by eosin staining and dehydration. The stained sections were then mounted on the slides using neutral balsam. Image acquisition of the sections was performed using an optical microscope.

### 4.15 Two‐Photon Calcium Imaging

We conducted two‐photon calcium imaging with a resonant‐galvo scanning microscope (Scientifica; 512 × 512 pixels; 30 fps), employing a 16×/0.8NA water‐immersion objective (Nikon). Excitation was provided by a mode‐locked Ti:sapphire laser (920 nm; 30–50 mW at the objective; Mai Tai eHP DeepSee, Spectra‐Physics, USA). The imaging field of view, roughly 250 × 250 µm^2^, was focused on the superior sagittal sinus, located 250–400 µm deep beneath the S2 pia mater. ScanImage software (Vidrio Technologies) was used to manage image acquisition.

### 4.16 Calcium Imaging Analysis

First, we used the MATLAB (MathWorks) EZcalcium toolbox to correct the two‐photon calcium signal video. Next, we manually delineated single‐neuron regions of interest (ROIs) in ImageJ and extracted their average fluorescence values. Finally, we applied a 5 Hz low‐pass filter and calculated ΔF/F as follows:

$$\Delta F/F=F_{t}-F_{0}/F_{0}$$

where F_t_ is the real‐time recorded fluorescence signal, while F_0_ is the average value between the 25th and 80th percentiles of the baseline fluorescence signal, calculated over a 3‐s period before the start of each stimulus. A neuron is considered responsive if its activity (ΔF/F) during the stimulation window differs significantly from the baseline values (measured 2 s before stimulus onset; *p* < 0.05, Wilcoxon signed‐rank test).

The individual cell trace data were processed using ΔF/F, with the baseline established as the 10 s before the first stimulation. Where F0 is the average between the 25th and 80th percentiles of the fluorescence signal at the baseline 10 s before the start of the first stimulus, and Ft is the fluorescence signal recorded in real time.

The correlation of neuron population activity was assessed by computing the Pearson correlation coefficients between the trial‐averaged responses of all recorded neurons to the identical stimulus across each stimulus.

Trial activity correlation was assessed using the Pearson correlation coefficient, which was based on the average responses of all recorded neurons during each trial of the same stimulus.

The neuron population activity correlation was assessed using the Pearson correlation coefficient, which was based on the average responses of each neuron recorded during the same stimulus period.

The mean neuronal response under different occlusal stimulation was calculated by determining the average response of each neuron during the first 1/3 of the stimulation period across five identical occlusal stimulation trials.

### 4.17 Clinical Evaluation on 3D‐Printed Dental Crowns

The clinical study was examined and approved by the Institutional Ethics Committee of Wuhan Union Hospital, Huazhong University of Science and Technology, and all the participants have signed the informed consent form before operation. A standard criteria for volunteer inclusion was set in detail: Age at 18–65; Dental implantation for molar or premolar missing for ≥3 months; Bone height ≥10 mm, bone width ≥6 mm (CBCT assessment post operation); Good oral hygiene (plaque index ≤20%, probing depth ≤3 mm). When included into experimental groups, basal clinic information was recorded in detail.

When it was time for placement of dental crowns (≥3 months post implantation), different types of dental crowns (ZrO_2_ crown and piezoelectric ZrO_2_/BCZT crown) were installed onto implants of recruited volunteers sequentially with a single‐blind principle, and they were required to perform indicated biting behaviors, after which their subjective sensational information were collected according to experimental design. In detail, with the patient's fully informed consent and under the careful guidance of an experienced clinician, we will first gently comfort and help the patient relax into a calm state. The patient will then be invited to try four distinct chewing methods: Mild Biting, Normal Biting, Tight Biting, Continuous Chewing. Each method will be performed 3 to 5 times under professional supervision. After each trial, we'll kindly ask the patient to mindfully compare the sensation:“How does the restored crown (whether on your Natural Tooth or Dental Implant) feel compared to your adjacent healthy natural teeth?” To help us understand patients’ experience precisely, please select the most accurate descriptor from these five sensory levels: No sensation, Mild sensation, Distinct sensation, Tolerable discomfort/pain, Intolerable pain. Patients’ feedback directly guides our care, and we'll continuously monitor their comfort throughout this process.

### 4.18 Statistical Analysis

Custom scripts developed in MATLAB (version 2020b, MathWorks) were used to conduct statistical analysis. For all datasets, statistical significance was defined as *p* < 0.05. In detail, we used the Wilcoxon signed‐rank test to define responsive cell population; a two‐sided two‐way repeated measures ANOVA for test mean response, cell correlation and trial correlation with post hoc Bonferroni corrections for multiple comparisons; two‐way repeated measures ANOVA to test cell culture time.

### 4.19 MTT Assay

Cell viability was determined by MTT Cell Proliferation and Cytotoxicity Assay Kit (Beyotime, China). Briefly, NIH/3T3 cells were seeded in 96‐well plates and incubated for 24 h, and cells were co‐cultivated with different concentrations of BCZT powders. After the treatments, cells were washed with phosphate‐buffered saline (PBS) and then incubated in MTT solution (Beyotime) for 4 h. After Formazan was added into each well and dissolved, the absorbance was measured at 570 nm to determine the cell viability with a microplate reader (BioTek, USA).

### 4.20 Calcein‐AM/Propidium Iodide (PI) Staining

Cell cytotoxicity of the PIT was assessed with NIH/3T3 cells cocultured with BCZT powders for 48 h using a Calcein AM/PI Double Stain Kit (MKBio, Shanghai, MX3012) following the manufacturer's protocols. After the preparation of the assay buffer and stain buffer, cells were incubated with the stain buffer at 37°C for 15 min. Fluorescent images were captured on a confocal microscope (Nikon A1‐Si).

## Conflicts of Interest

The authors declare no conflicts of interest.

## Supporting information




**Supporting File**: advs74942‐sup‐0001‐SuppMat.docx.

## Data Availability

The data that support the findings of this study are available on request from the corresponding author. The data are not publicly available due to privacy or ethical restrictions.
