# Supplementary material for: An Implanted Tooth That Can Feel
Source: Adv Sci (Weinh). 2026 Mar 23;13(32):e20786. doi: 10.1002/advs.202520786 (PMC13252652; doi:10.1002/advs.202520786)
Supplement: Supplementary file 1 — Supporting File: advs74942‐sup‐0001‐SuppMat.docx. [file ADVS-13-e20786-s001.docx]

**Supplementary Figures**


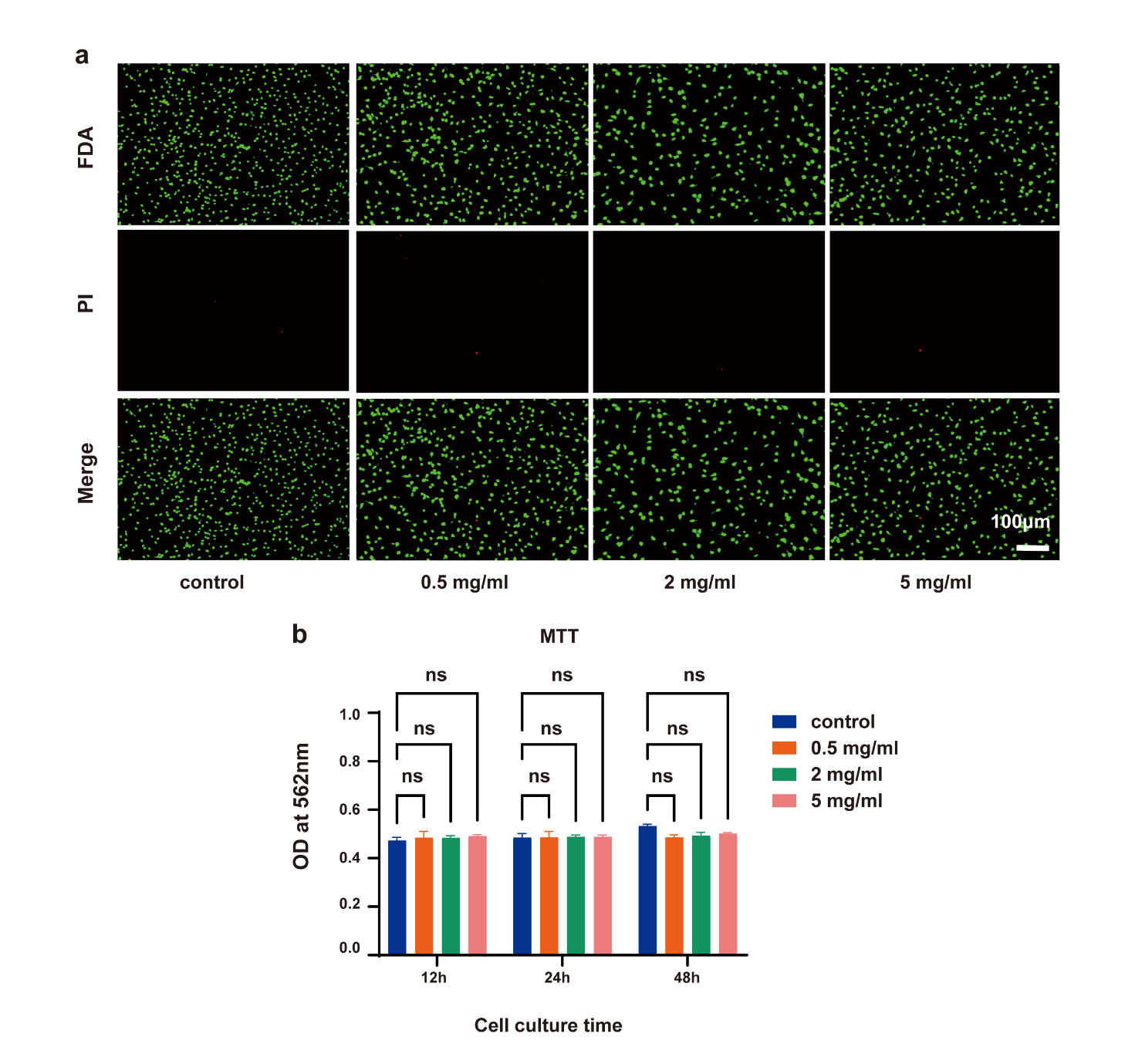


**Figure S1. The PIT exhibits a long working life and reliability.** In vitro biocompatibility evaluation. (a) Live/Dead staining of 3T3-L1 cells seeded in culture medium mixed with different concentrations of materials. Live cells are stained green while apoptotic cells are stained red. (b) Viability of 3T3-L1 cells cultured in above culture medium measured by MTT assay. Data analyzed by (b, n=5) two-way repeated measures ANOVA .


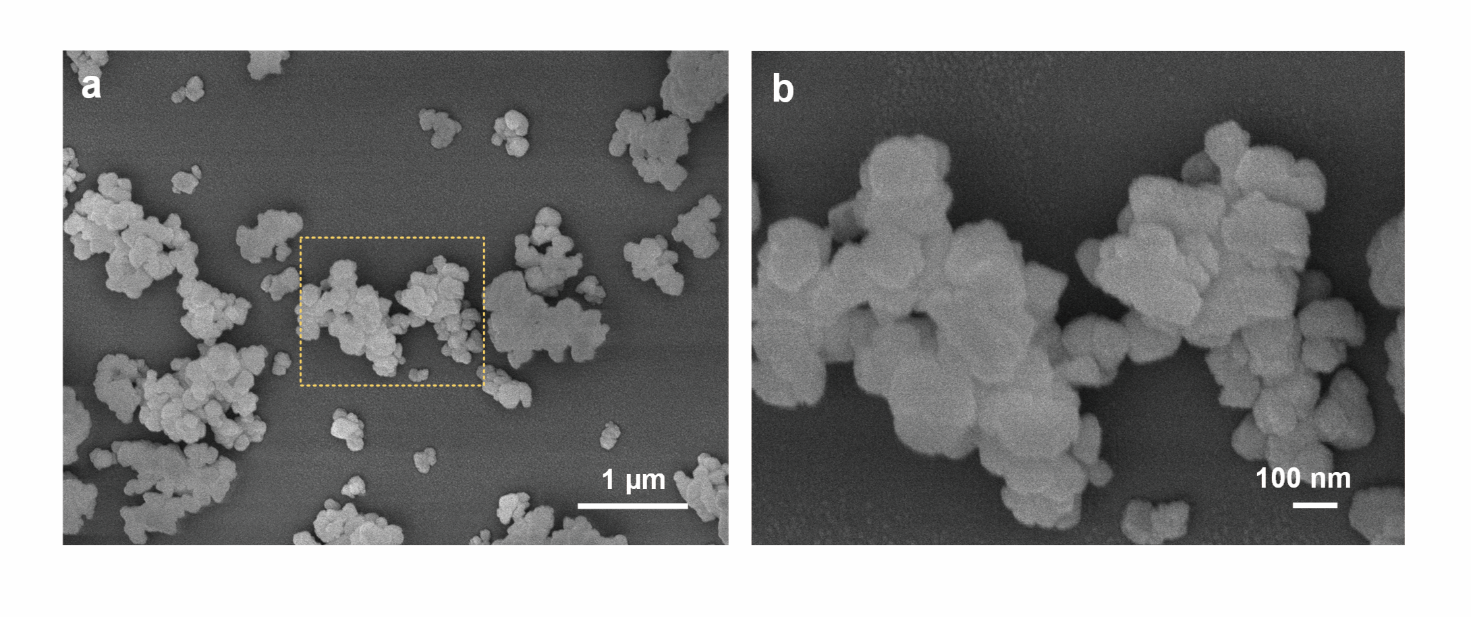


**Figure S2.** (a) SEM image of zirconia (ZrO_2_) powders for 3D printing; (b) is magnified image of (a).


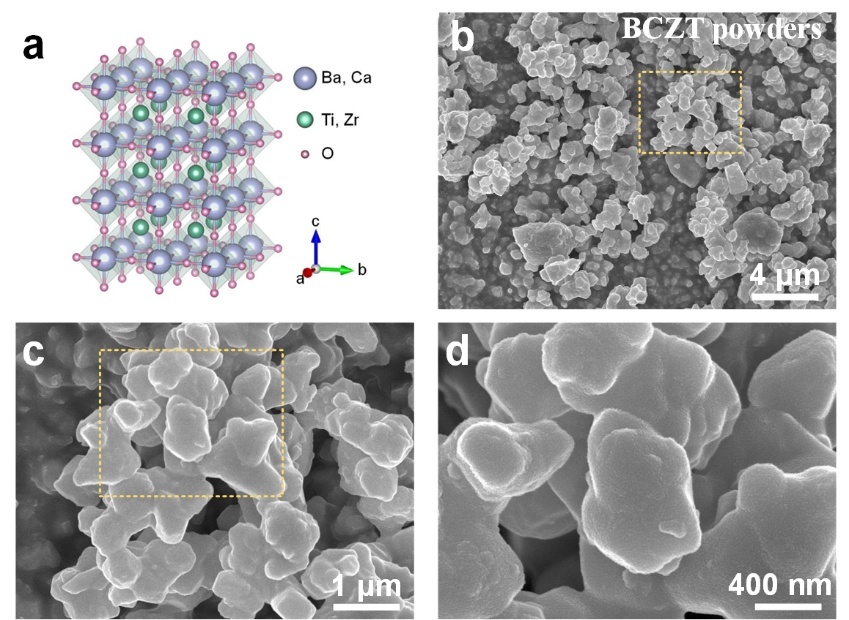


**Figure S3.** (a) Schematic illustration of molecular structures of barium calcium zirconate titanate (BCZT); (b) SEM image of BCZT powders for 3D printing; (c) is magnified image of (b); (d) is magnified image of (c).


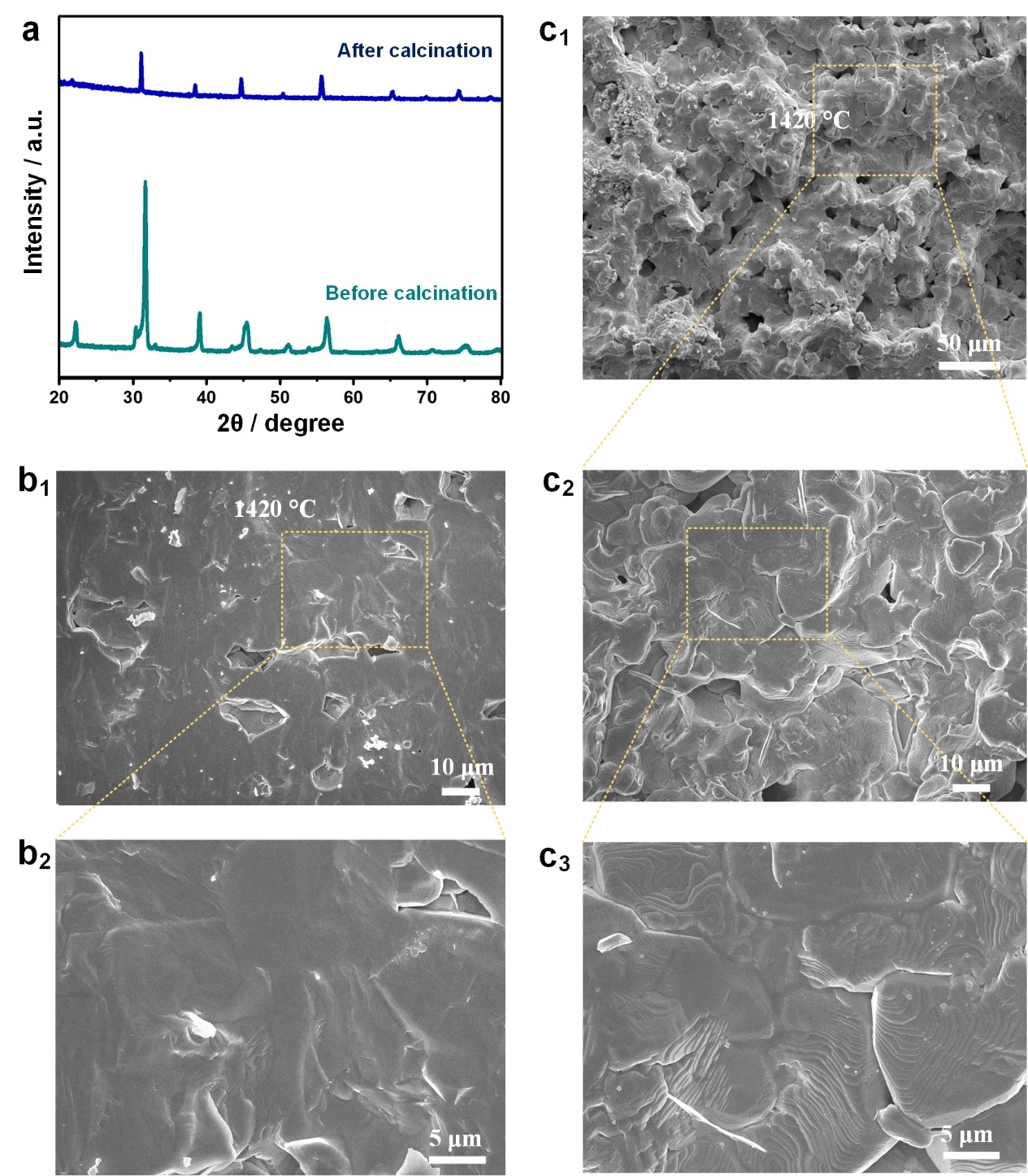


**Figure S4.** **BCZT characterization.** (a) X-ray diffraction (XRD) of a 3D-printed BCZT scaffold before (bottom) and after (top) the calcination. (b_1-2_) Top view and (c_1-3_) cross-sectional view SEM images of a calcined BCZT scaffold. The bottom images are magnified ones.


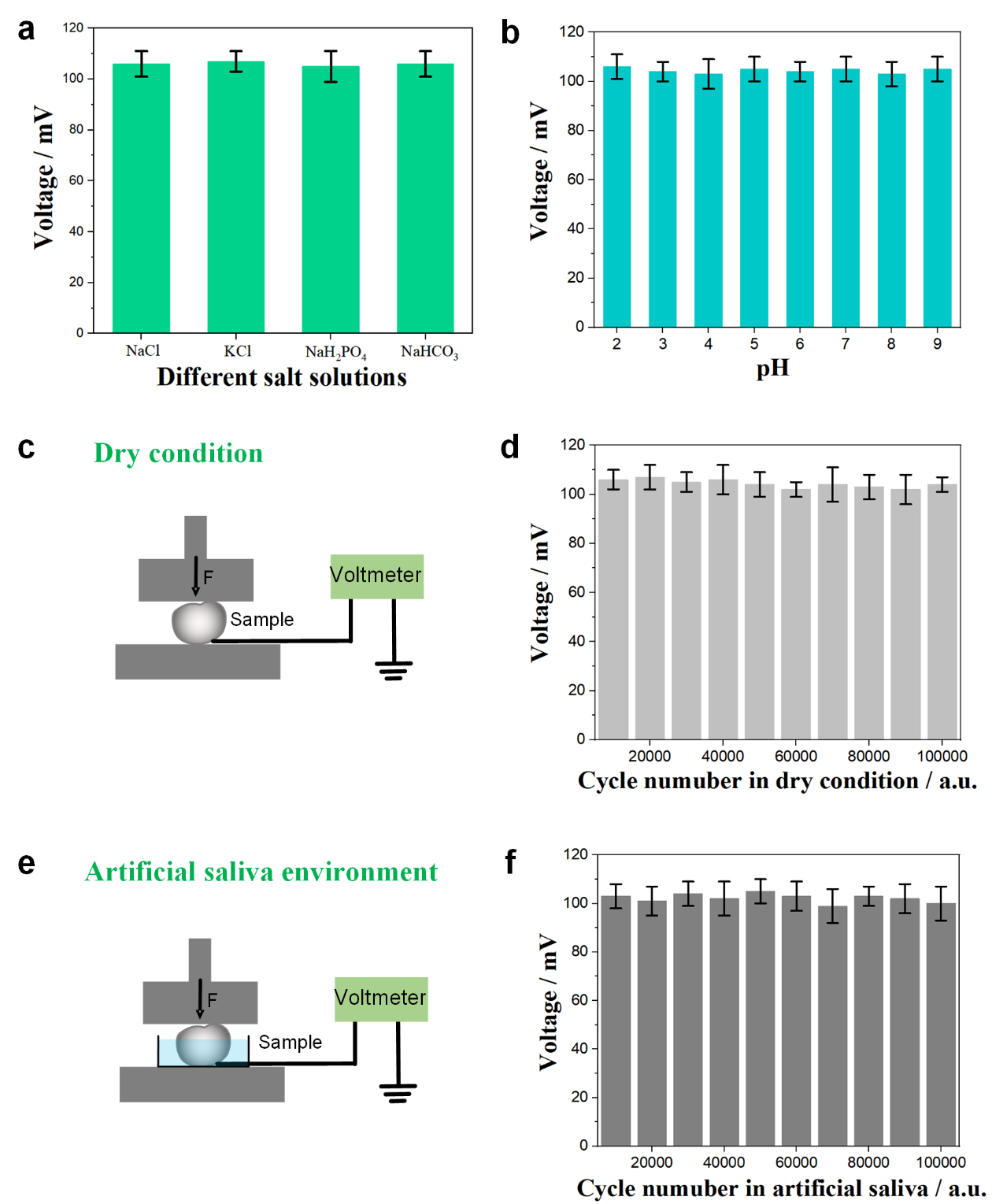


**Figure S5.** **The stability tests of the electrical signal output of a PIT**. (a) The statistical analysis of the electrical signal output of a PIT in different salt solution environments. The concentrations of the solutions are all 0.1 mol/L. (b) The statistical analysis of the electrical signal outputs of a PIT in different pH solution environments. (c-f) The 100,000-cycle long-term tests of a PIT in dry environments and saliva environments, respectively.


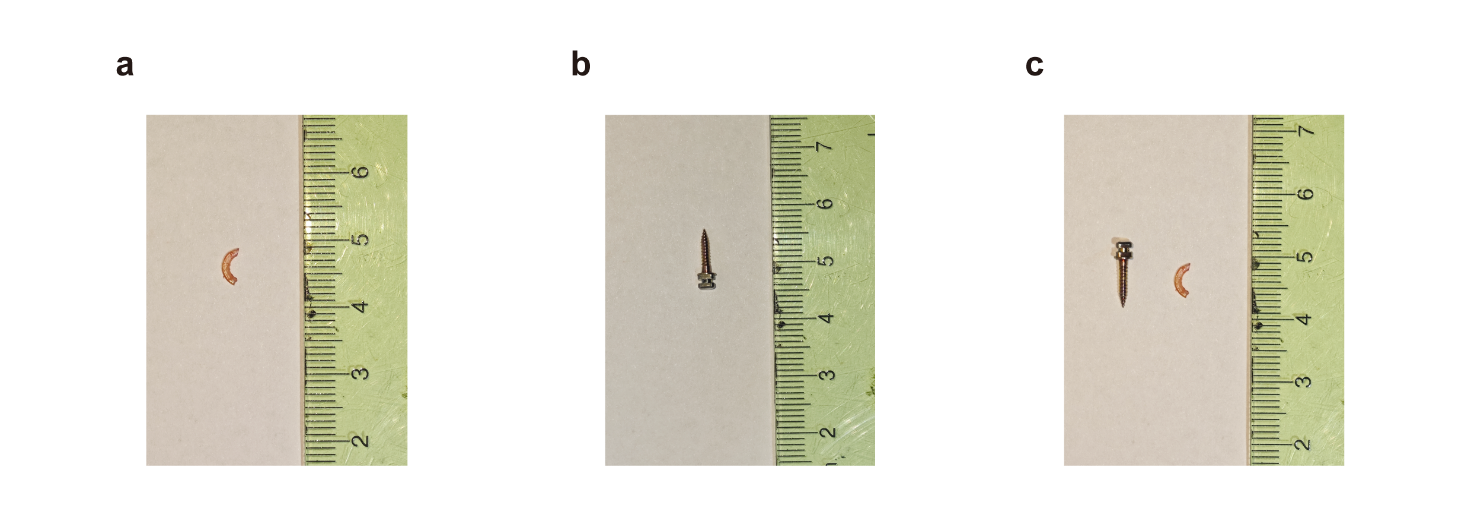
**Figure S6. Optical photographs of (a) extracted incisor of experimental mice and (b, c) loaded metal implants to extracted sites.**


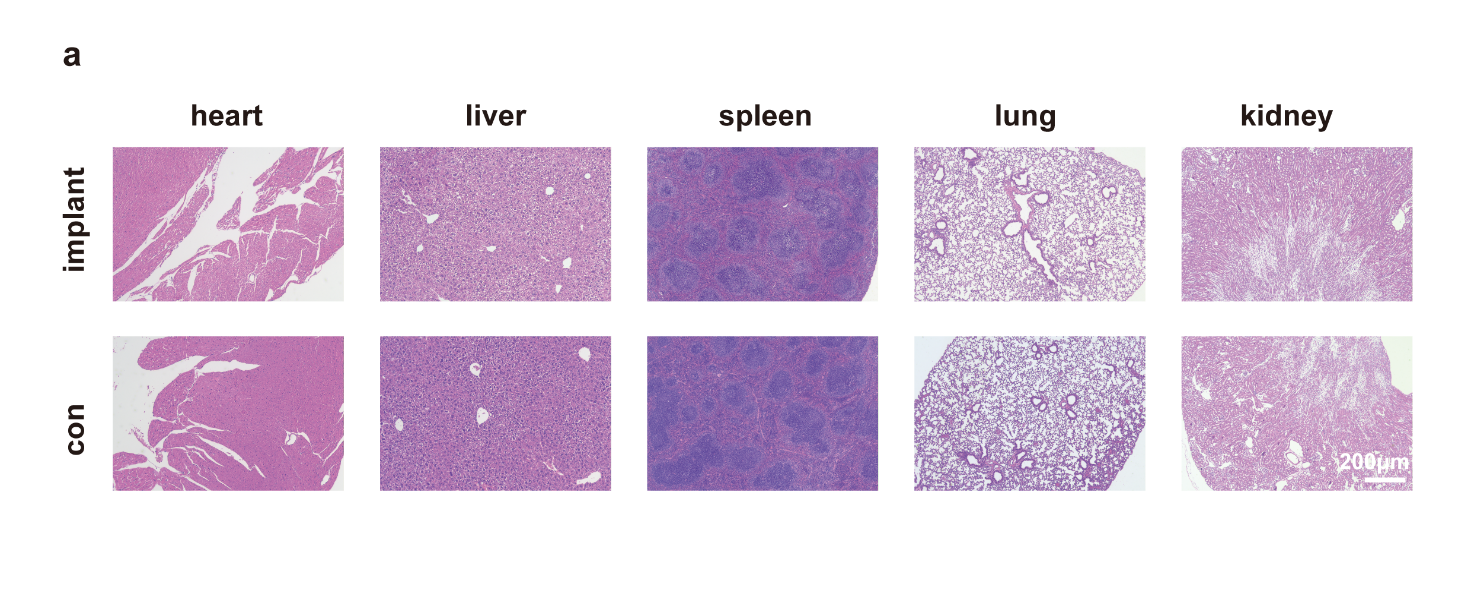
**Figure S7. In vivo biocompatibility evaluation of internal organs after implantation operation.**


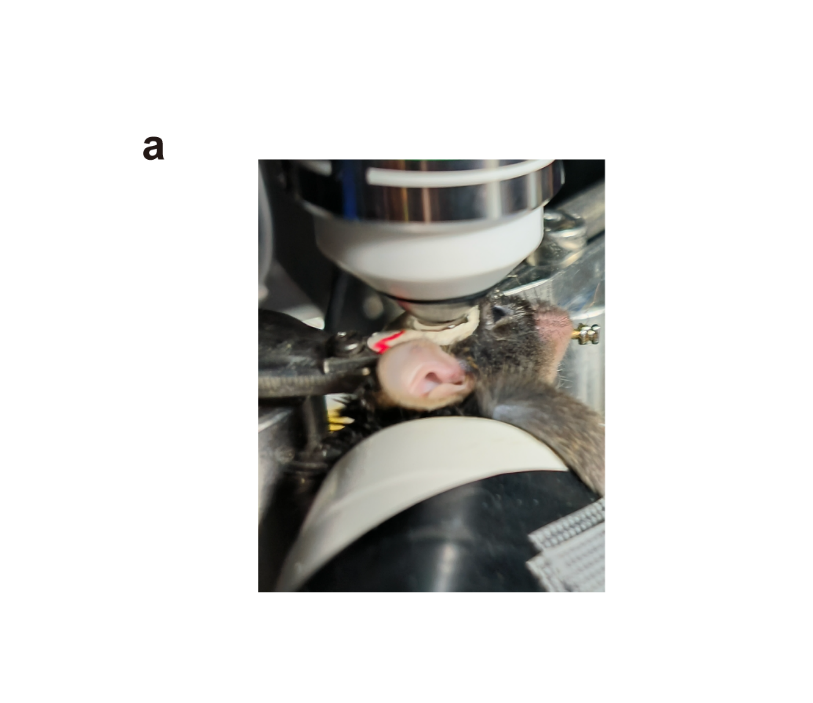
**Figure S8. Optical photograph of image capturing of head-fixed mice using two-photon microscopy.**

**
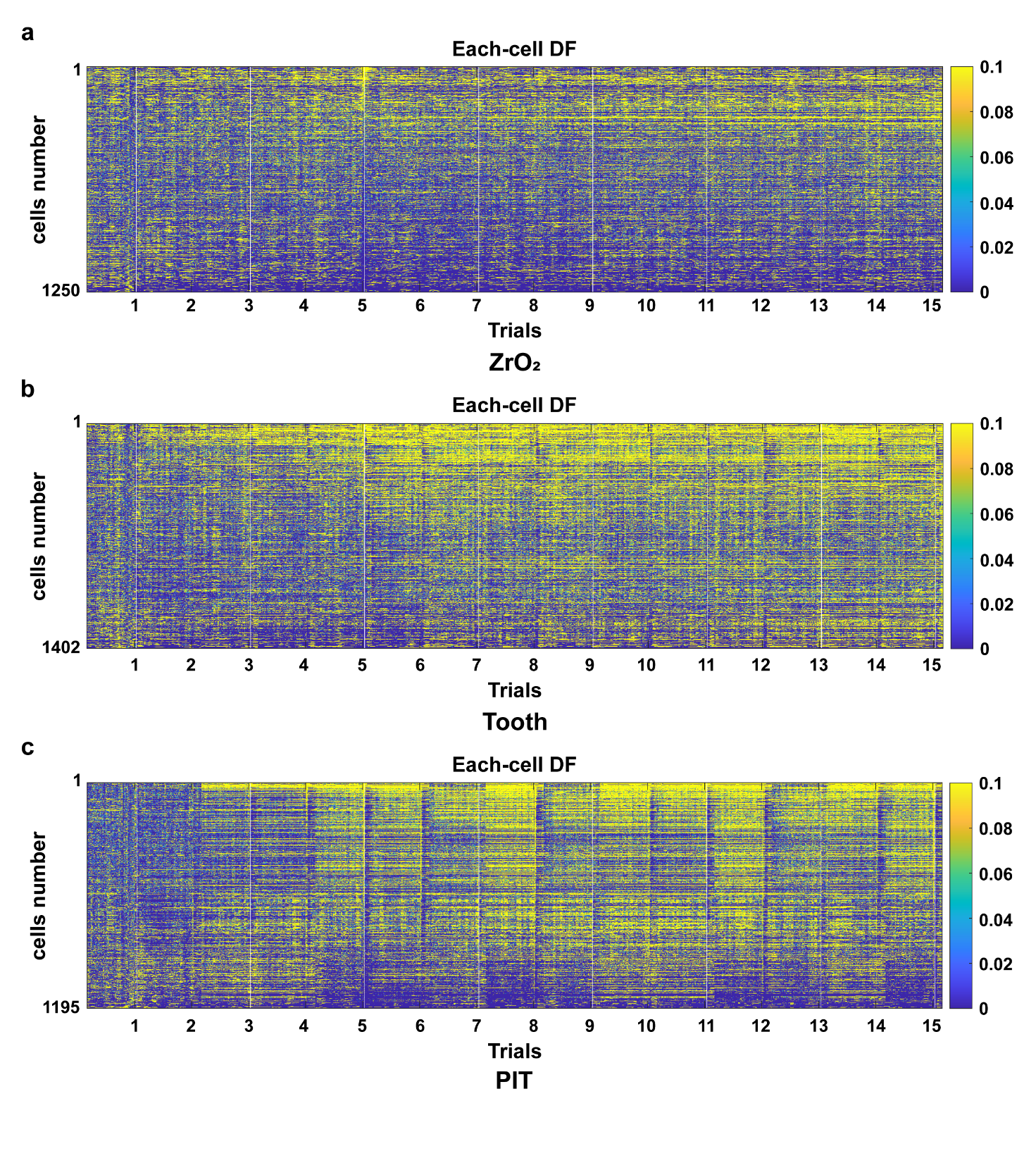
**


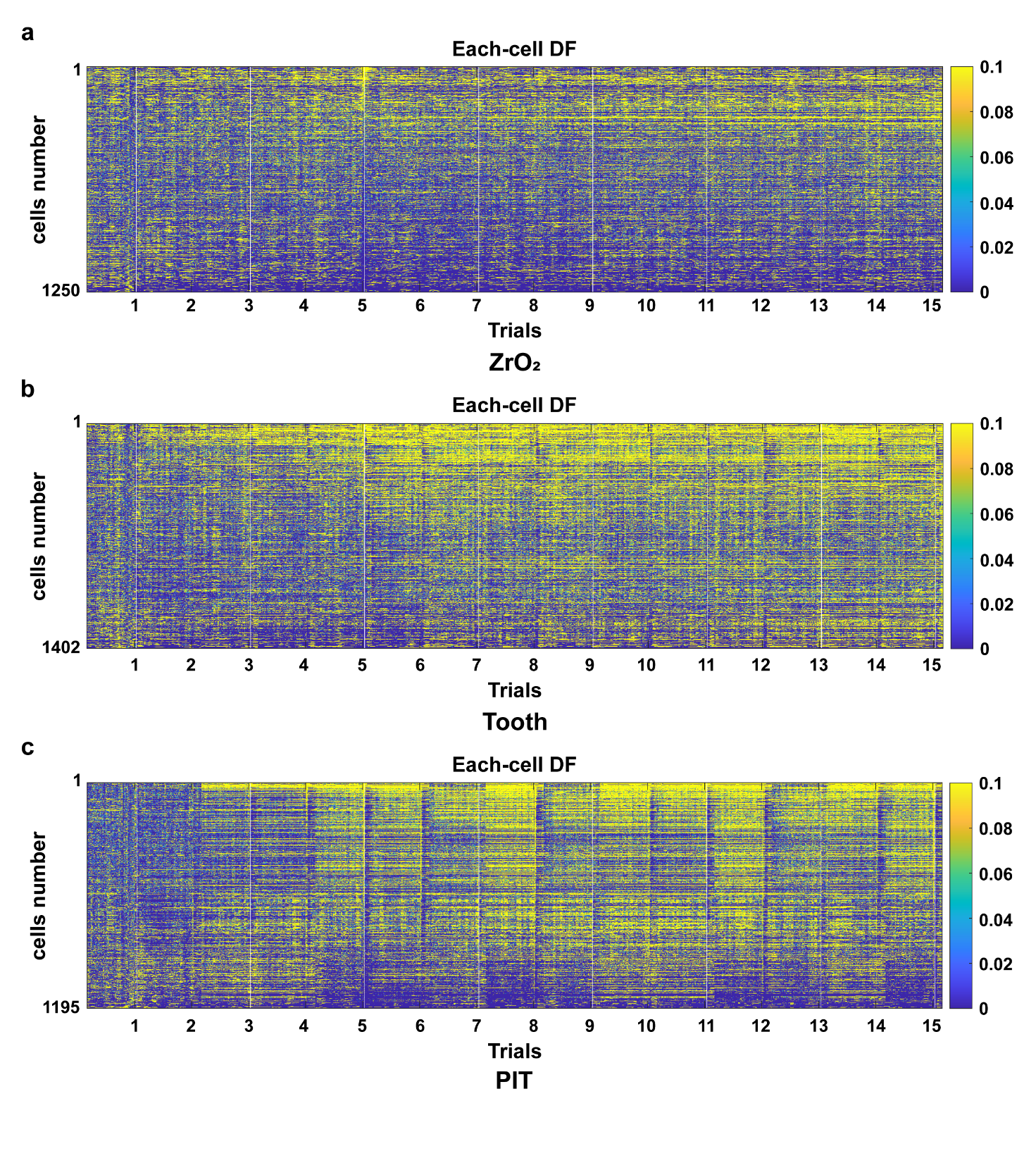
**Figure S9. Heat maps of all neuron activities in response to multiple occlusal loadings from the ZrO_2_, natural tooth, and PIT groups (ZrO_2_ (*n* = 7 mice), tooth (n=7 mice), PIT (n=8 mice)).**

**
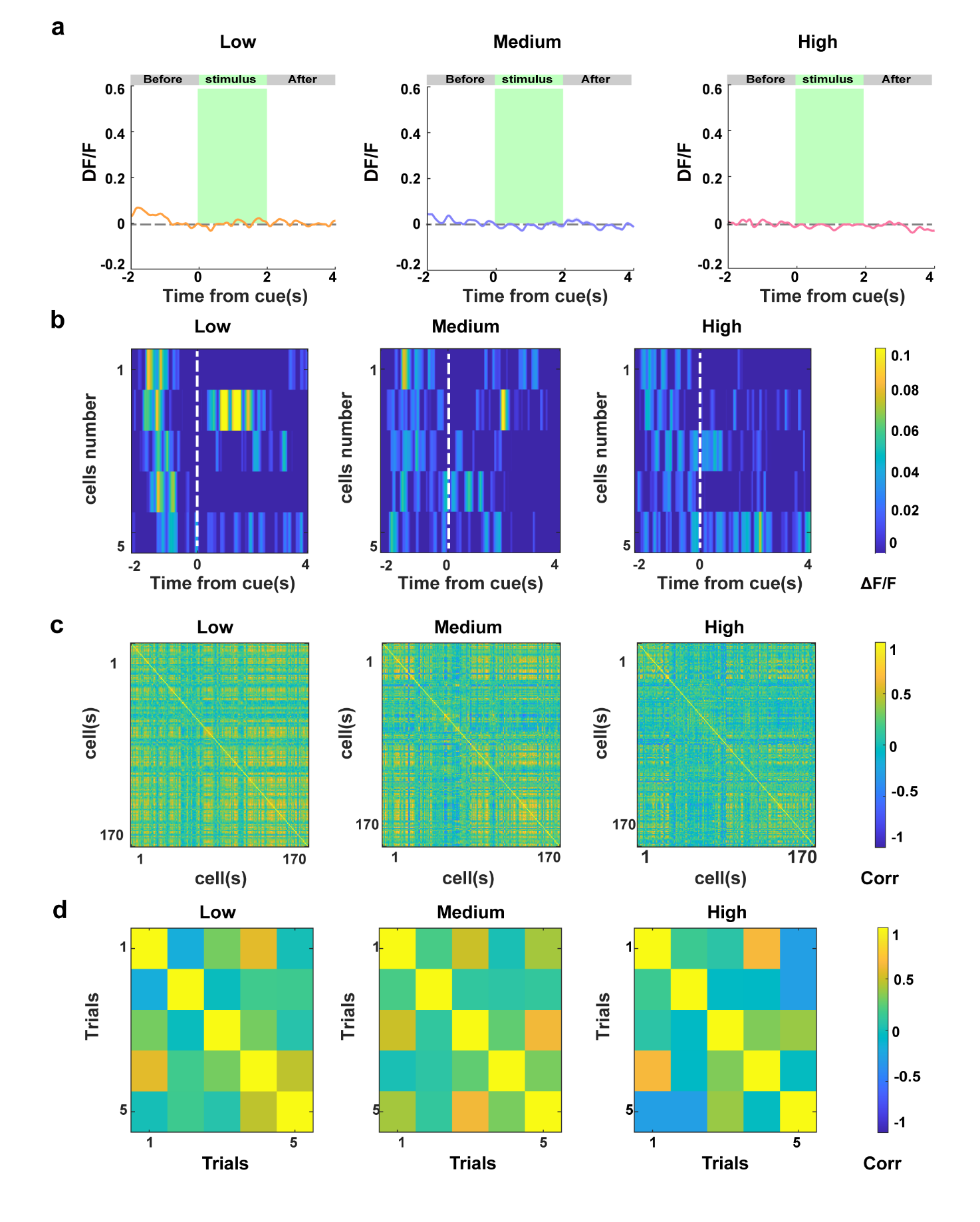
**

**Figure S10. Two-photon imaging data recorded from this example mouse during occlusal stimulation** **from the ZrO_2_ group.** (a) Trail-averaged neural response amplitude of recorded S2 neurons from representative experimental mice in response to low, medium and high levels of occlusal loadings (mean ± s.e.m). (b) Heat maps of neural activities of recorded S2 in response to occlusal loadings in each trail. (c) The neuron activity correlation of S2 neurons from all trials in response to occlusal stimulation at different levels. (d) The trial activity correlation of S2 neurons from all neurons in response to occlusal stimulation at different levels.


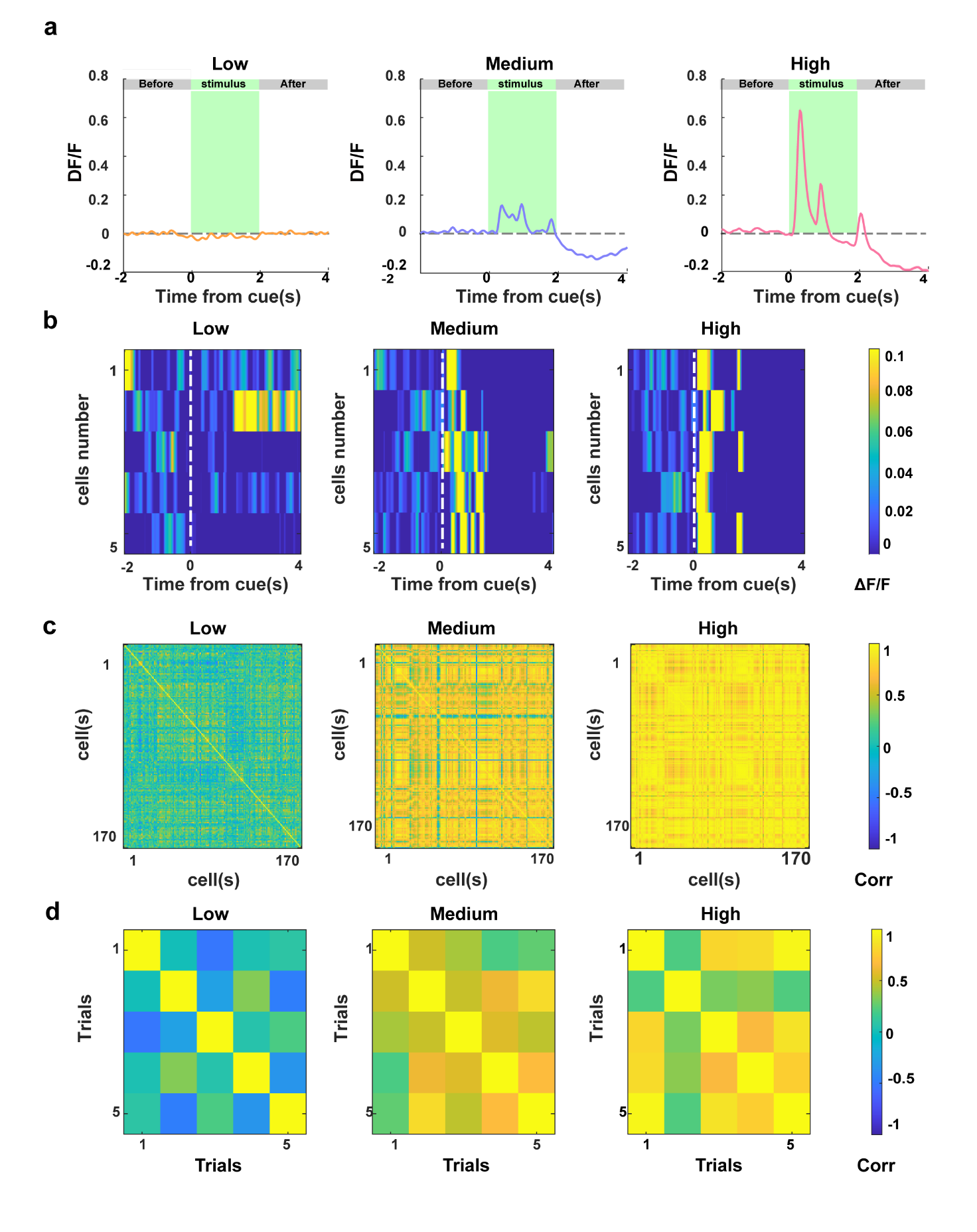


**Figure S11. Two-photon imaging data recorded from this example mouse during occlusal stimulation** **from the tooth group.** (a) Trail-averaged neural response amplitude of recorded S2 neurons from representative experimental mice in response to low, medium and high levels of occlusal loadings (mean ± s.e.m). (b) Heat maps of neural activities of recorded S2 in response to occlusal loadings in each trail. (c) The neuron activity correlation of S2 neurons from all trials in response to occlusal stimulation at different levels. (d) The trial activity correlation of S2 neurons from all neurons in response to occlusal stimulation at different levels.


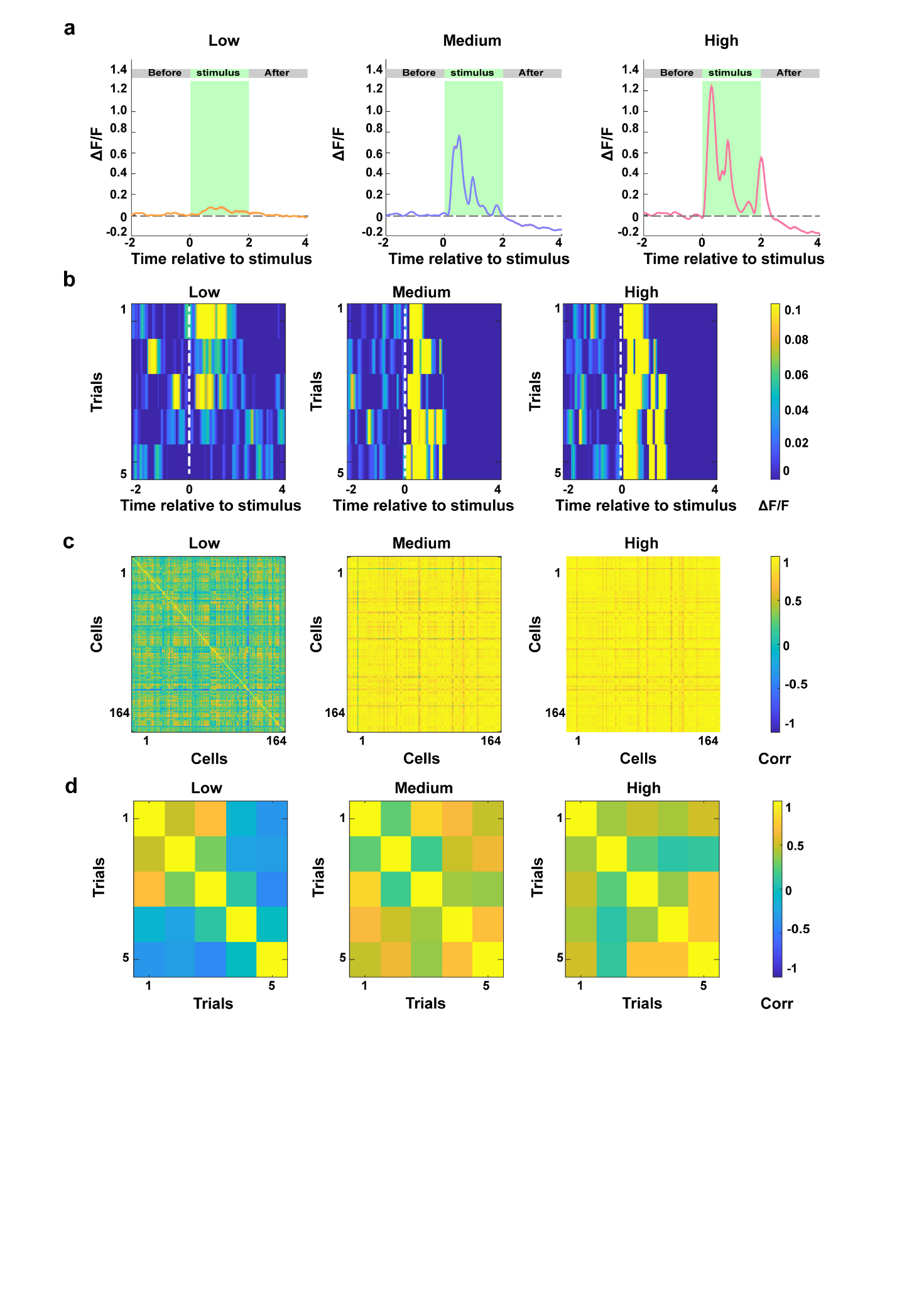


**Figure S12. Two-photon imaging data recorded from this example mouse during occlusal stimulation from the PIT group.** (a) Trail-averaged neural response amplitude of recorded S2 neurons from representative experimental mice in response to low, medium and high levels of occlusal loadings (mean ± s.e.m). (b) Heat maps of neural activities of recorded S2 in response to occlusal loadings in each trail. (c) The neuron activity correlation of S2 neurons from all trials in response to occlusal stimulation at different levels. (d) The trial activity correlation of S2 neurons from all neurons in response to occlusal stimulation at different levels.


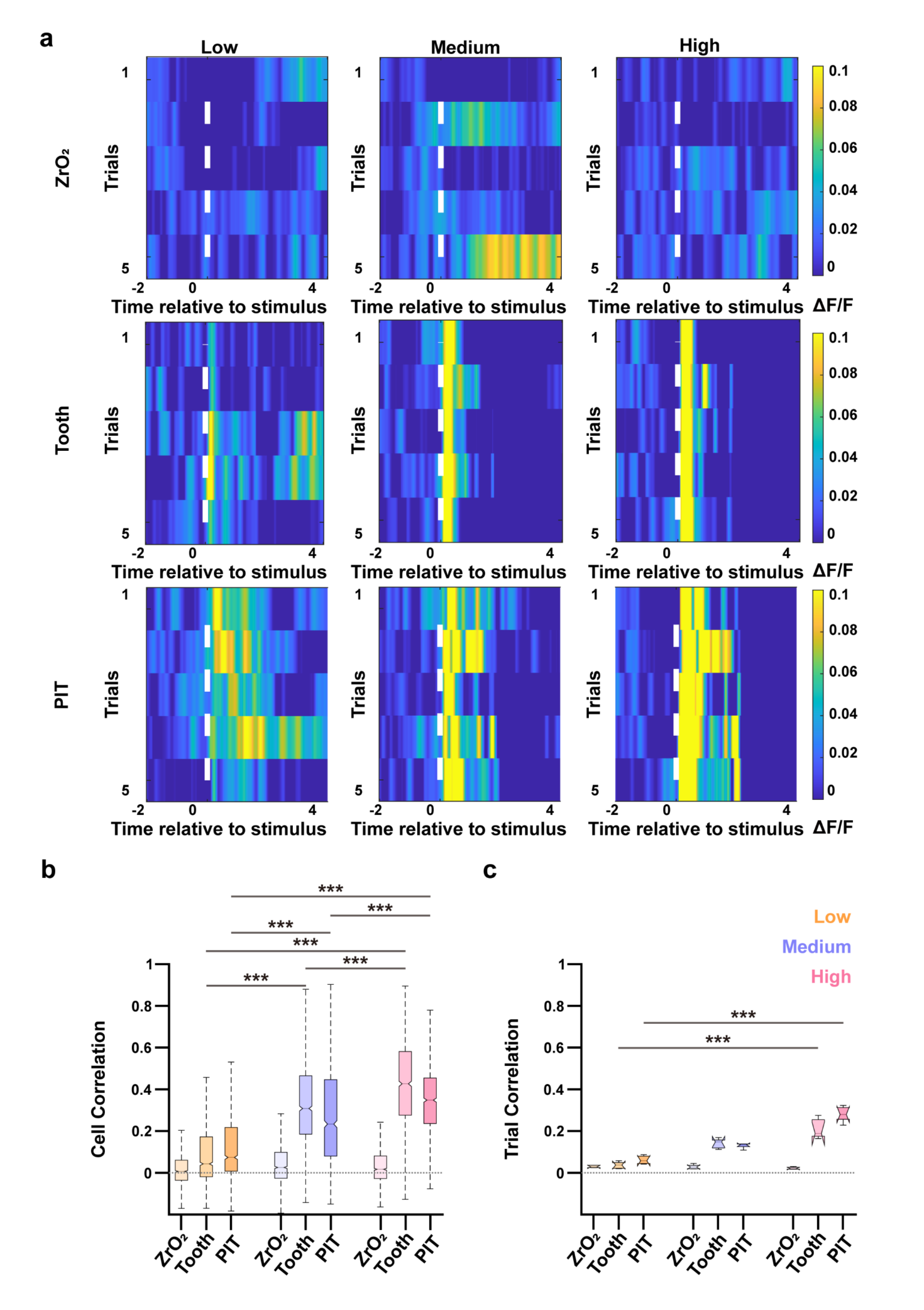


**Figure S13. Heatmap of all neuronal responses recorded from S2 during different occlusal stimuli, along with the correlation analysis of cells and trials**. (a) The top row of panel A represents the heatmaps of trail-averaged response of recorded all S2 neurons during occlusal stimulation at different levels from the ZrO_2_ group (*n* = 7 mice). The middle row of panel A represents the heatmaps of trail-averaged response of recorded all S2 neurons during occlusal stimulation at different levels from the tooth group (*n* = 7 mice). The down row of panel A represents the heatmaps of trail-averaged response of recorded all S2 neurons during occlusal stimulation at different levels from the PIT group (*n* = 8 mice). (b) The Population-averaged neuron activity correlation of S2 neurons from all trials in response to occlusal stimulation at different levels from the ZrO_2_, natural tooth, and PIT groups. (c) The Population-averaged trial activity correlation of S2 neurons from all neurons in response to occlusal stimulation at different levels from the ZrO_2_, natural tooth, and PIT groups. Data analyzed by (**b, c,** (ZrO_2_ (*n* = 7 mice), tooth (n=7 mice), PIT (n=8 mice)) two-sided two-way repeated measures ANOVA for analysis with post-hoc Bonferroni comparisons. Data are presented as (**b, c,** box plots (center line, median; box limits, upper and lower quartiles; whiskers, 1.5 × interquartile range).


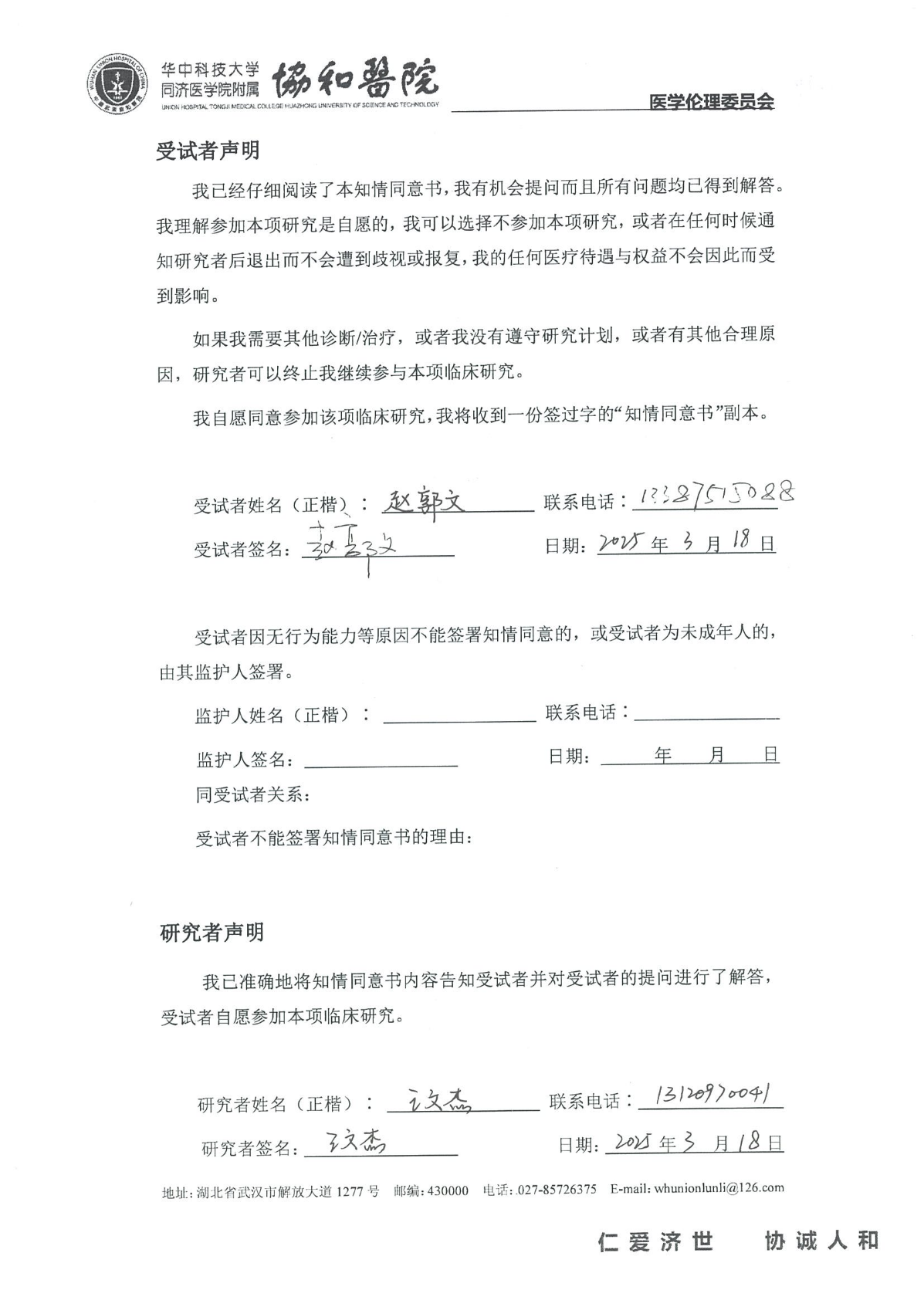


**Figure S14. Example of the personal informed content for volunteers.**

**Table S1**. **Baseline characteristics of the recruited volunteers.**

| Clinical Index | Values |
| --- | --- |
|  |  |
| Age [Y，M (Range)] | 44 (22-60) |
| Gender |  |
| Male | 11 (44.6) |
| Female | 12 (42.2) |
| Periodontal status [n (%)] |  |
| Plaque index |  |
| <1 | 8 (34.8) |
| 1-2 | 15 (65.2) |
| Odontolith index |  |
| 0 | 1 (4.3) |
| 0-1 | 16 (69.6) |
| ≥1 | 6 (26.1) |
| Gingiva color |  |
| 0 | 23 (100) |
| Gingiva texture |  |
| 0 | 21 (91.3) |
| 0-1 | 2 (8.7) |
| Gingiva recession |  |
| 0 | 18 (78.3) |
| 0-1 | 5 (21.7) |
| Oral mucosa status [n (%)] |  |
| Ulcer |  |
| 0 | 23 (100) |
| Fibrosis |  |
| 0 | 23 (100) |
| Leukoplakia |  |
| 0 | 23 (100) |
| lichen planus |  |
| 0 | 23 (100) |
| Implant status [n (%)] |  |
| Probe depth |  |
| 0 | 23 (100) |
| Probe bleeding |  |
| 0 | 23 (100) |
| Probe pyorrhea |  |
| 0 | 23 (100) |
| CBCT examination |  |
| 0 | 23 (100) |
|  |  |
